# Transcriptomic Profiling and H3K27me3 Distribution Reveal Both Demethylase-Dependent and Independent Regulation of Developmental Gene Transcription in Cell Differentiation

**DOI:** 10.1371/journal.pone.0135276

**Published:** 2015-08-11

**Authors:** Sung Chul Kang, Se Kye Kim, Jin Choul Chai, Sun Hwa Kim, Kyoung-Jae Won, Young Seek Lee, Kyoung Hwa Jung, Young Gyu Chai

**Affiliations:** 1 Department of Nanobiotechnology, Hanyang University, Seoul, Republic of Korea; 2 Department of Molecular and Life Science, Hanyang University, Ansan, Republic of Korea; 3 Institute of Diabetes, Obesity, and Metabolism, Genetics Department, Smilow Center for Translational Research, Perelman School of Medicine, University of Pennsylvania, Philadelphia, Pennsylvania 19104, United States of America; Università degli Studi di Milano, ITALY

## Abstract

The removal of histone H3 trimethylation at lysine residue 27 (H3K27me3) plays a critical role in the transcriptional initiation of developmental genes. The H3K27me3-specific KDM6 demethylases JMJD3 and UTX are responsible for the transcriptional initiation of various developmental genes, but some genes are expressed in a KDM6 demethylase-independent manner. To address the role of H3K27me3 in the retinoic acid (RA)-induced differentiation of the human carcinoma NCCIT cell line, we inhibited JMJD3 and UTX using the H3K27me3 demethylase inhibitor GSK-J4. The commitment of JMJD3/UTX-inhibited cells to a specific fate was delayed, and transcriptome profiling also revealed the differential expression of genes related to cell fate specification in demethylase-inactivated cells; the expression levels of RA metabolism and HOX family genes significantly decreased. We observed a weak correlation between H3K27me3 enrichment and transcriptional repression in the control and JMJD/UTX-inhibited cells, except for a few sets of developmental genes that are indispensable for cell fate specification. Taken together, these results provide the H3K27me3 landscape of a differentiating cell line and suggest that both demethylase-dependent and demethylase-independent transcriptional regulation play a role in early differentiation and developmental gene expression activated by H3K27me3 demethylation.

## Introduction

The differentiation of pluripotent stem cells requires dramatic changes in the initiation and suppression of gene transcription to accomplish cell specification. Numerous modifications modulate the regulation of gene expression programs, including post-translational histone modifications. These covalent modifications epigenetically regulate and maintain lineage-specific gene expression during development. Two specific histone modifications play a critical role in the regulation of developmental genes: a repressive marker, the trimethylation of histone H3 on lysine residue 27 (H3K27me3), and an activating marker, the trimethylation of histone H3 on lysine residue 4 (H3K4me3). These two modifications co-exist at promoters that regulate the expression of essential developmental genes in embryonic stem (ES) cells and other progenitor cell lines, forming epigenetic signatures called bivalent promoters [1–3]. Due to the co-existence of these modifications, promoters are “poised” for gene activation, rapidly responding to developmental stimuli. Bivalent promoters can change their histone modification state to either an H3K4me3-dominant (active) or an H3K27me3-dominant (inactive) state, and this state primarily depends on H3K27me3 demethylation [4]. Previous reports showed that H3K27me3 demethylation is required for gene activation in various cell types [4–9], implying that the precise regulation of H3K27me3 demethylation must be maintained for proper development.

The Jumonji-C domain (JmjC)-containing histone demethylase family KMD6 is responsible for H3K27me3 demethylation. Three KDM6 demethylases, JMJD3 (KDM6B), UTX (KDM6A) and UTY, can remove one methyl residue from H3K27me3 and H3K27me2; however, the activity of UTY is significantly lower than that of other demethylases [10–13]. JMJD3 and UTX play an essential role in differentiation by changing compact heterochromatin structures to open states, allowing poised promoters to be activated by the recruitment of lineage-specific transcription factors. JMJD3 promotes epithelial-mesenchymal transition in murine epithelial cells [14]. In mouse embryonic stem cells, JMJD3 regulates neural marker expression, thereby mediating neural commitment [4]. Knockout and knockdown studies of JMJD3/UTX suggested that these demethylases play an essential role in the development of the central nervous system [15–17], respiratory system [6, 18] and cardiac system [19]. Whereas H3K27me3 acts as a suppressive marker and H3K27me3 demethylases have been highlighted as regulatory factors in differentiation, recent reports have indicated weak correlations between H3K27me3 and demethylases in cell types that previously showed JMJD3/UTX activity and H3K27me3 during cellular development. JMJD3/UTX-knockout mouse zygotes exhibited a normal lifespan or displayed developmental defects during the late stage of differentiation, surviving the early lethality that is expected due to defects in demethylase-dependent cell commitment. UTX has been shown to mediate embryonic development, mesoderm induction and differentiation in a demethylase-independent fashion [11, 20, 21]. At the molecular level, JMJD3 and UTX play a demethylase-independent chromatin remodeling role in murine EL4 cells and primary T cells [22]. A recent report states that H3K27me3 demethylation during early embryonic development may occur in a KDM6 demethylase-independent manner [23]. Despite the pivotal role of their catalytic function, these recent findings suggest complex roles for JMJD3 and UTX in cell commitment.

Previous studies have assessed the significance of JMJD3/UTX functions in biological processes using several techniques to disable the catalytic activities of these enzymes, including RNA-interference techniques and site-specific mutagenesis [4, 9, 20, 24]. Although these approaches are readily applied in the field, they may affect the integrity of the demethylases, unintentionally hindering their other transcriptional regulatory functions. A solution to this undesired manipulation of enzymatic integrity is the design of a chemical inhibitor that binds to the active site, thereby preventing the interaction between JMJD3/UTX and H3K27me3 without compromising the conformation of these enzymes. GSK-J4 is an ethyl ester derivative of GSK-J1, a JMJD3/UTX-selective histone demethylase inhibitor [25]. Since its development, GSK-J4 has been applied in various studies of the function of JMJD3 and its role in various biological processes [26–28]. In this work, we also used GSK-J4 as a JMJD3/UTX inhibitor to study the role of H3K27me3 in the retinoic acid (RA) induced differentiation of human carcinoma NCCIT cells. We observed differences in the transcriptome of wild-type and H3K27me3 demethylation-inhibited NCCIT cells during differentiation; the simple inhibition of H3K27me3-specific demethylases could delay cell specification. We also investigated the distribution of this suppressive histone modification and identified changes in the levels of H3K27me3 at the promoters of developmental genes due to JMJD3/UTX inhibition, whereas differences in other genes were insignificant. The presented results provide a global view of demethylase-dependent and demethylase-independent transcription during early development.

## Materials and Methods

### Cell culture conditions and RA treatment

Human embryonal carcinoma (EC) NCCIT (ATCC, Manassas, VA) cells were cultured in RPMI 1640 and OPTIMEM media (Invitrogen, Carlsbad, CA) to maintain and differentiate the cells, respectively. All media were supplemented with 100 U/ml penicillin, 100 μg/ml streptomycin (Invitrogen) and 10% heat-inactivated fetal bovine serum (Thermo Scientific, Waltham, MA). Embryonic bodies (EB) were formed as previously described [29]. All-trans RA (Sigma-Aldrich, St. Louis, MO) was prepared in DMSO. After growing for 24 h, the EBs were treated with RA or RA supplemented with GSK-J4 (Tocris Bioscience, Bristol, United Kingdom) and cultured in 5% CO_2_ at 37°C for 48 h. EBs treated with RA and RA plus GSK-J4 are hereafter referred to as EB_RA_ and EB_RA+GSK_, respectively.

### JMJD3 knockout NCCIT cell line construction using a CRISPR-Cas9 system

JMJD3 knockout NCCIT cells were constructed using the clustered regularly interspaced short palindromic repeats (CRISPR)/CRISPR-associated 9 (Cas9) system based on a method designed by Mali *et al*. [30], with modifications. To prepare JMJD3 guide RNA (gRNA) fragments, four sites in a *JMJD3*-coding region were amplified by polymerase chain reaction (PCR) using four sets of primers (Table 1). The gRNA cloning vector (Addgene plasmid ID 41824, Cambridge, MA) was linearized using AflII (New England Biolabs, Ipswich, MA), and mixed with four gRNA inserts separately for ligation by Gibson Assembly method using the Gibson Assembly Master Mix (New England Biolabs) according to the manufacturer’s instructions. NEB 5-α competent cells (New England Biolabs) were then transformed with the ligated DNAs, and the constructed gRNA vectors were purified and sequenced for confirmation. The purified gRNA vectors and the pCas9-GFP vector (Addgene plasmid ID 44719) were then mixed at a molar ratio of 20:1 and transfected into NCCIT cells using Lipofectamine LTX and PLUS Reagent (Invitrogen) according to the manufacturer’s instructions. Cells expressing green fluorescent protein 48 h post-transfection were sorted, plated on RPMI-containing dishes at a density of 3000–5000 cells per dish, and allowed to grow for up to two weeks. Visible colonies were then transferred to 24-well plates and allowed to grow for genomic DNA extraction. The *JMJD3* knockout in NCCIT cells was further confirmed by PCR, sequencing using primers flanking the target regions (Table 1) and quantitative real-time PCR (qRT-PCR) for *JMJD3* mRNA expression.

**Table 1:** Oligonucleotide primer sets used in this study.

	Primer Sequence (5’ ~ 3’)	
	Forward	Reverse
**JMJD3 gRNA1**	TTTCTTGGCTTTATATATCTTGTGGAAAGGACGAAACACCGAAGCTGCCCGCCTTCATGC	GACTAGCCTTATTTTAACTTGCTATTTCTAGCTCTAAAACGCATGAAGGCGGGCAGCTTC
**JMJD3 gRNA2**	TTTCTTGGCTTTATATATCTTGTGGAAAGGACGAAACACCGTGGCTCAGCATGTTGCCCG	GACTAGCCTTATTTTAACTTGCTATTTCTAGCTCTAAAACCGGGCAACATGCTGAGCCAC
**JMJD3 gRNA3**	TTTCTTGGCTTTATATATCTTGTGGAAAGGACGAAACACCGCTCGTCCTTGACACGGCCC	GACTAGCCTTATTTTAACTTGCTATTTCTAGCTCTAAAACGGGCCGTGTCAAGGACGAGC
**JMJD3 gRNA4**	TTTCTTGGCTTTATATATCTTGTGGAAAGGACGAAACACCGCTGGAGCAGTACCGCACTG	GACTAGCCTTATTTTAACTTGCTATTTCTAGCTCTAAAACCAGTGCGGTACTGCTCCAGC
**JMJD3(1–2)-1**	GTTCCTGCTTCCTTCCCCTC	TCACAGAAAGCGCTGATGGT
**JMJD3(1–2)-2**	TGATGCTAAGCGGTCAGTGG	GTCTCCCAGTAGTGCTCGTG
**JMJD3(3–4)-3**	CCTCTGCCCTTGCTCCAG	TAGGTCCAGCCCAGGCAT
**JMJD3(3–4)-4**	TGGTCTCAACACAACCCCAC	CCTCATCGCGACGTGCT
**NANOG**	AGCTACAACAGGYGAAGAC	GGTGGTAGGAAGAGTAAAGG
**POU5F1**	TCTATTTGGGAAGGATT	ATTGTTGTCAGCTTCCTCCA
**SOX2**	TCCCATCACCCACAGCAAATGA	TTTCTTGTCGGCATCGCGGTTT
**NES**	TGGCGCACCTCAAGATGTC	GGTCCTAGAATTGCAGCTC
**PAX6**	AGATTCAGATGAGGCTCAAA	AATTGGTTGGTAGACACTGG
**BMP4**	GCTGAGGTTAAAGAGGAAACGA	TGGTCTTGAGTATCCTGAGCG
**RBP1**	CAACTGGCTCCAGTCACTCC	TGCACGATCTCTTTGTCTGG
**STRA6**	CCACAGAGGACTACTCCTATGG	CAGCACAAGGATTGACAGCG
**CRABP1**	CAGGACGGGGATCAGTTCTA	CGCCAAACGTCAGGATAAGT
**CRABP2**	ATCGGAAAACTTCGAGGAATTGC	AGGCTCTTACAGGGCCTCC
**CYP26A1**	CATGTTCTCCAGAAAGTGCG	GGGATTCAGTCGAAGGGTCT
**GAPDH**	ATGGGGAAGGTGAAGGTCG	GGGGTCATTGATGGCAACAATA

### RNA preparation and qRT-PCR

Total RNA samples were extracted from EB_RA_ and EB_RA+GSK_ by homogenization in RNAiso Plus, and the extracts were further purified according to the manufacturer’s instructions (TaKaRa BIO, Shiga, Japan). First-strand cDNA was synthesized with SuperScript II Reverse Transcriptase (Invitrogen). qRT-PCR was performed with the synthesized cDNA, SYBR Premix Ex Taq II and the appropriate primers (Table 1) using the ABI 7500 Real-Time PCR system (Applied Biosystems, Carlsbad, CA), as previously described [31].

### Western blotting

Whole cell extracts were prepared in RIPA buffer [50 mM Tris-Cl, pH 7.5; 150 mM sodium chloride; 0.5% sodium deoxycholate; 0.1% sodium dodecyl sulfate; 1% (v/v) Nonidet P-40] supplemented with *cOmplete* EDTA-free Protease Inhibitor Cocktail (Roche, Mannheim, Germany). Total proteins were separated on polyacrylamide gels by sodium dodecyl sulfate polyacrylamide gel electrophoresis (SDS-PAGE) and transferred to polyvinylidene difluoride (PVDF) membranes (Schleicher & Schuell Bioscience, Inc., Keene, NH). After blocking with PBS supplemented with 5% horse serum (Invitrogen) for 2 h, the membranes were blotted with the appropriate primary antibodies at 4°C for 16 h, followed by blotting with horseradish peroxidase-conjugated anti-rabbit immunoglobulin G (IgG; Jackson ImmunoResearch Laboratories, Inc., West Grove, PA; 711-545-152) secondary antibody for 1 h at room temperature. After washing with TBS-T (20 mM Tris-Cl, pH 7.6; 500 mM NaCl; 0.1% Tween 20), the reactions were detected using ECL Prime Western Blotting Detection Reagent (GE Healthcare, Wauwatosa, WI).

The following antibodies were used for western blotting and were all purchased from Abcam (Cambridge, the United Kingdom): anti-histone H3 (ab1791), anti-H3K27me3 (ab6002) and anti-histone H3 dimethylated lysine 27 (H3K27me2, ab24684).

### KDM6 demethylase activity inhibition assay

Nuclear extracts were prepared using an EpiQuik Nuclear Extraction Kit (Epigentek, Farmingdale, NY) according to the manufacturer’s instructions. The inhibition of JMJD3/UTX in NCCIT, EB_RA_ and EB_RA+GSK_ was examined with an Epigenase JMJD3/UTX Demethylase Activity/Inhibition Colorimetric Assay Kit (Epigentek) using the nuclear extracts (20 μg) according to the manufacturer’s instructions. The inhibition ratio was measured in triplicate, and the percentage of inhibition was calculated based on three different assays.

### Chromatin immunoprecipitation (ChIP) and ChIP-qPCR

NCCIT cells were collected and resuspended in digestion buffer (50 mM Tris-Cl, pH 7.6; 1 mM CaCl_2_, 0.2% Triton X-100, 5 mM butyrate, 1X protease inhibitor cocktail, 0.5 mM PMSF), followed by incubation with 0.3 U Micrococcal nuclease (MNase; Sigma-Aldrich) at 37°C for 5 min. The reaction was stopped with 50 mM EDTA and treated with RIPA buffer for 16 h, followed by incubation with an appropriate antibody (see below) and Dynabeads Protein A beads (Invitrogen) for 16 h at 4°C. The chromatin-antibody-Dynabeads Protein A complexes were consecutively washed with RIPA buffer supplemented with 0.3 M NaCl, lithium chloride (LiCl) buffer and TE buffer, and then incubated with proteinase K at 65°C for 16 h. The DNA from the complexes was purified via phenol/chloroform extraction and concentrated by ethanol precipitation. The resulting DNA pellets were dissolved in TE buffer for further analyses.

ChIP-qPCR was performed similarly to qPCR, with one modification: the cDNA was replaced with immunoprecipitated DNA. The primers used in ChIP-qPCR are listed in Table 1. The following antibodies were used for the ChIP analysis and were purchased from Abcam, Santa Cruz Biotechnology (Dallas, TX) and Cell Signaling (Danvers, MA): control IgG (Santa Cruz sc-2025), anti-H3K27me3 (Cell Signaling #9733), anti-RNA polymerase II (RNAPII, Abcam ab5131) and anti-H3K4me3 (ab8580).

### RNA sequencing (RNA-seq) and ChIP sequencing (ChIP-seq)

Ribosomal RNAs (rRNAs) were removed from total RNA using the RiboMinus Transcriptome Isolation Kit (Invitrogen). rRNA-depleted total RNAs were used to construct paired-end transcriptome libraries using the NEBNext Ultra Directional RNA Library Prep Kit for Illumina (New England Biolabs). Briefly, first-strand cDNAs were synthesized from rRNA-depleted RNA samples, followed by second-strand synthesis with DNA polymerase I and RNase H. The double-stranded cDNAs were then end-repaired and ligated to adaptors. The ligated libraries were then separated on a 2% agarose gel (Duchefa, Haarlem, The Netherlands), and fragments with sizes between 300–400 bp were purified using the MinElute Gel Extraction Kit (Qiagen, Hilden, Germany). The fragments were amplified for further enrichment and purified by ethanol precipitation. Two biological replicates were prepared from each condition.

Immunoprecipitated DNA was used to construct paired-end ChIP-sequencing libraries using the NEBNext Ultra DNA Library Prep Kit for Illumina. ChIP reactions using rabbit IgG and from untreated and RA-treated NCCIT were used as normalization controls.

The paired-end deep sequencing of the constructed libraries was performed using HiSeq 2000 and 2500 (Illumina, San Diego, CA) by the National Instrumentation Center for Environmental Management (NICEM, Seoul, Republic of Korea) and the Next-Generation Sequencing Core from the University of Pennsylvania (http://ngsc.med.upenn.edu/).

### Data analysis and statistics

For transcriptome expression profiling, the raw reads were trimmed using the FASTX-Toolkit [32] and aligned against USCS *Homo sapiens* hg19 using Tophat (version 2.0.10) [33]. The aligned reads were assembled with the Cufflinks package, version 2.2.1 [34]. Differentially expressed genes (DEGs) were identified using Cuffdiff [35]. All data were normalized according to their fragments per kilobase per million map reads (FPKM) for each gene [36]. DEGs displaying more than twofold changes in their log_2_ fold-change were selected for functional annotation using the Database for Annotation, Visualization and Integrated Discovery (DAVID) version 6.7 [37, 38] and the following parameters: threshold count 5 and enrichment probability (EASE score) 0.1. The raw reads used in this work were deposited into the Gene Expression Omnibus database (GEO, http://www.ncbi.nlm.nih.gov/geo) under accession number SRP045949.

For the ChIP-seq analysis, the raw reads were aligned using Bowtie2 version 2.2.1 [39]. UCSC hg19 was used as the reference index. Significantly enriched regions were identified using the hypergeometric Optimization of Motif EnRichment (HOMER) analysis package [40]. Default parameters were used for peak calling. The identified peaks were visualized using the UCSC Genome Brower (https://www.genome.ucsc.edu).

The JMJD3/UTX inhibition assay and qPCR data were statistically analyzed using SPSS 21.0 (SPSS Inc., Chicago, IL). All experiments were conducted in triplicate, and data are presented as the means ± SE. The data were tested with a one-way ANOVA followed by Tukey’s HSD post hoc test.

## Results

### The effect of GSK-J4 on the cellular morphology and the global H3K27me3 level in the early differentiation of NCCIT cells

**Fig 1:**
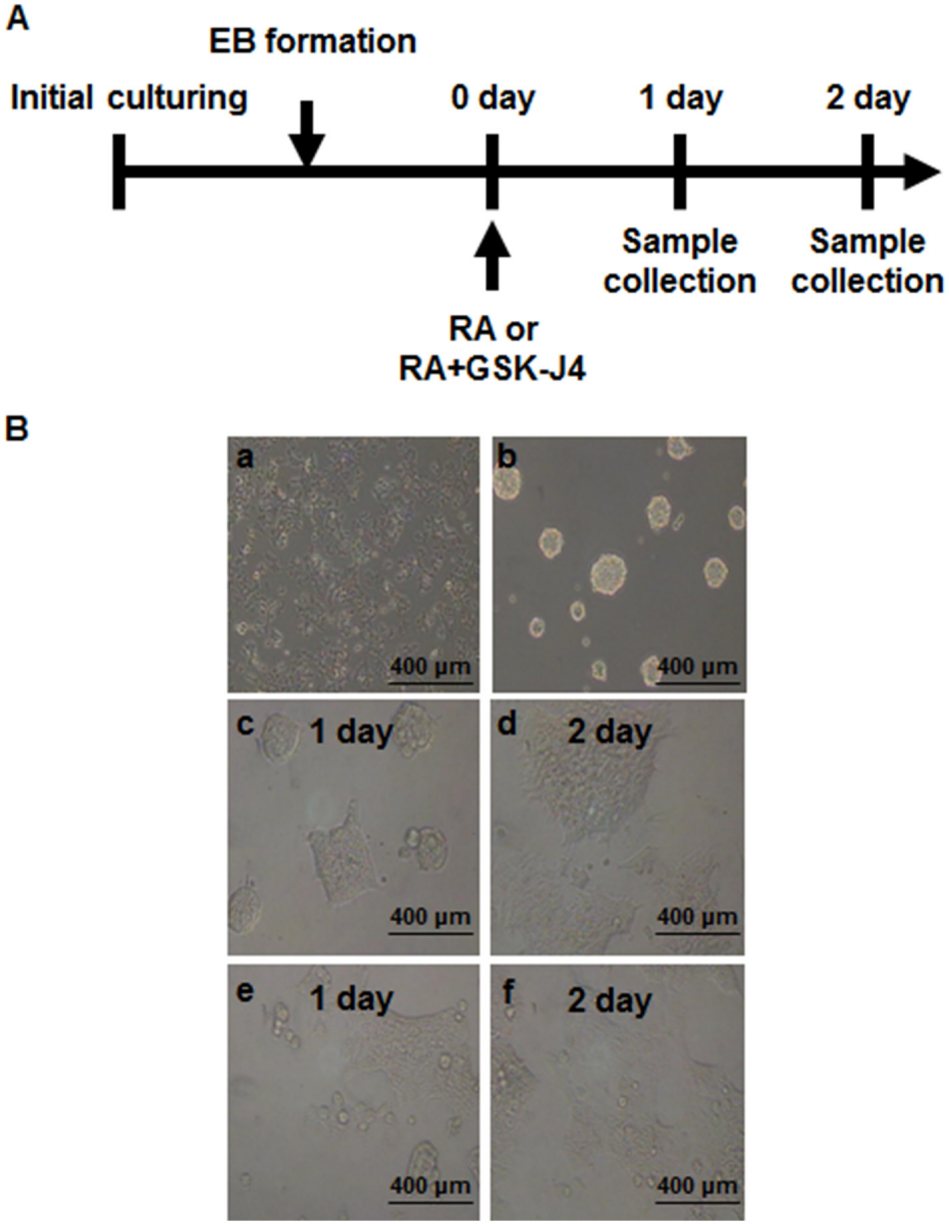
The effect of GSK-J4 on RA-induced differentiation. (A) Schematic illustration of RA and GSK-J4 treatment to NCCIT cells. NCCIT cells were stabilized and subcultured to form EBs. After stabilization, the EBs were treated with RA or RA+GSK-J4. The samples were then collected 1 and 2 days thereafter for analyses. (B) Cell morphology of NCCIT (a), EB (b), EB (c, 1 day; d, 2 day) and EB_RA+GSK_ (e, 1 day; f, 2 day).

**Fig 2:**
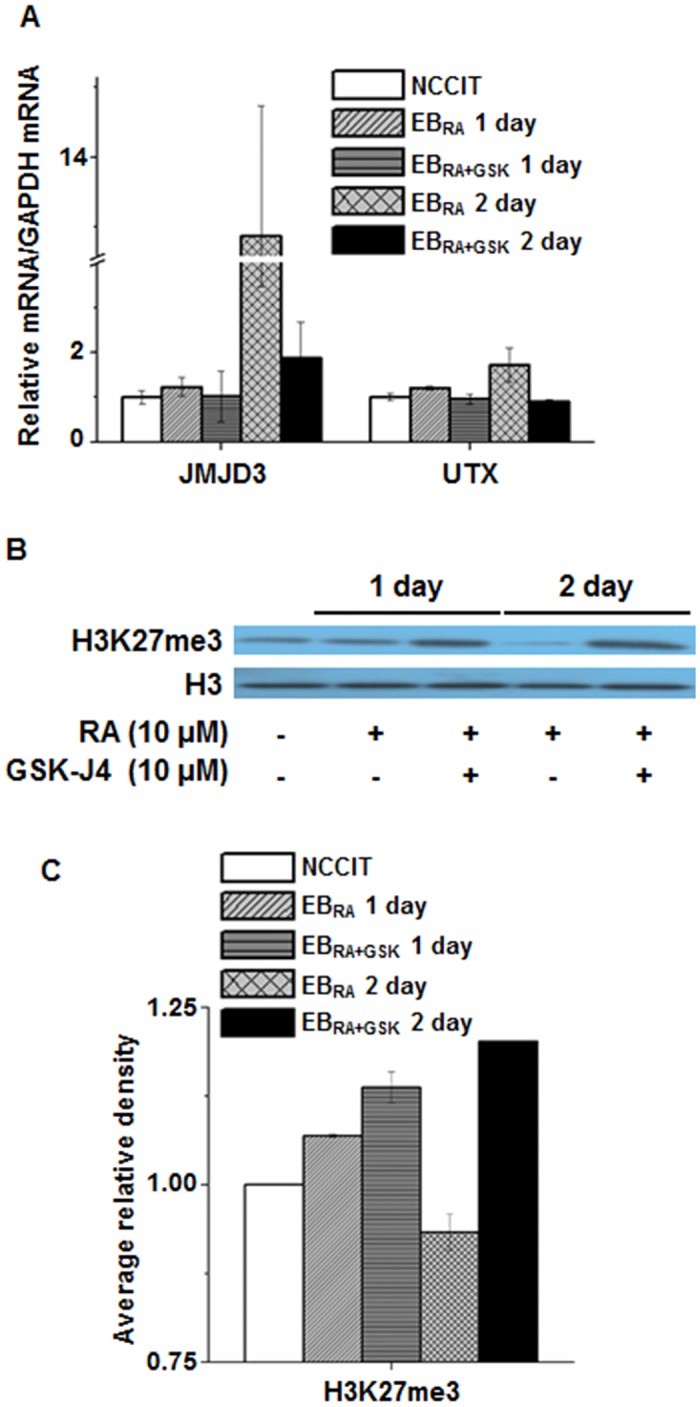
The effect of GSK-J4 on global H3K27me3 during RA-induced differentiation. (A) mRNA expression levels of KDM6 demethylases JMJD3 and UTX in RA-induced differentiation and GSK treatment. qRT-PCR data were normalized to GAPDH expression. The values are presented as the means ± SE (n = 3). (B) Changes in H3K27me3 level by GSK-J4 during NCCIT differentiation. Whole cell extracts were collected from cells treated for 1 day and 2 days, isolated using RIPA buffer, and immunoblotted with the following antibodies: H3 and H3K27me3. The experiment was performed in triplicate, and a representative blot image is shown. (C) Densitometric analysis of changes in H3K27me3 level by GSK-J4 during NCCIT differentiation. The average relative density of H3K27me3 was compared with the relative density of histone H3 based on triplicate experiments. The asterisk represents the significant difference analyzed by one-way ANOVA followed by Tukey’s HSD post hoc test (*: *P* < 0.05).

In this study, we aimed to specify the role of KDM6 demethylases during differentiation. NCCIT cells were developed into EBs, treated with either RA alone or RA supplemented with GSK-J4, and collected 24 and 48 h after the treatments (Fig 1A). After EB formation, the morphological changes of cells in response to RA-induced differentiation were assessed. Cell attachment and differentiation could be observed in EB_RA_ cells1 and 2 days after RA treatment. However, treating both RA and GSK-J4 partially inhibited cell attachment and delayed the differentiation of EB_RA+GSK_ cells (Fig 1B and S2 Fig). The transcriptional levels of JMJD3 and UTX were measured to assess the effect of GSK-J4 on the expression of KDM6 demethylases. JMJD3 and UTX expression increased during differentiation, and JMJD3 expression was greater than UTX expression. Upon GSK-J4 treatment, the expression of both KDM6 demethylases was decreased (Fig 2A). H3K27me3 levels in EB cells were also affected by GSK-J4 treatment: H3K27me3 was demethylated during differentiation, but GSK-J4 treatment reversed this reaction (Fig 2B). The effect of GSK-J4 on KDM6 demethylases was further confirmed using an inhibition assay, which showed that the amount of demethylated products was lower in in EB_RA+GSK_ cells than in EB_RA_ cells This difference implies that GSK-J4 inhibits the demethylase activities of JMJD3 and UTX and that the inhibitor delays the RA-induced differentiation of NCCIT.

### Transcriptomic profiling of NCCIT cells during early differentiation

**Fig 3:**
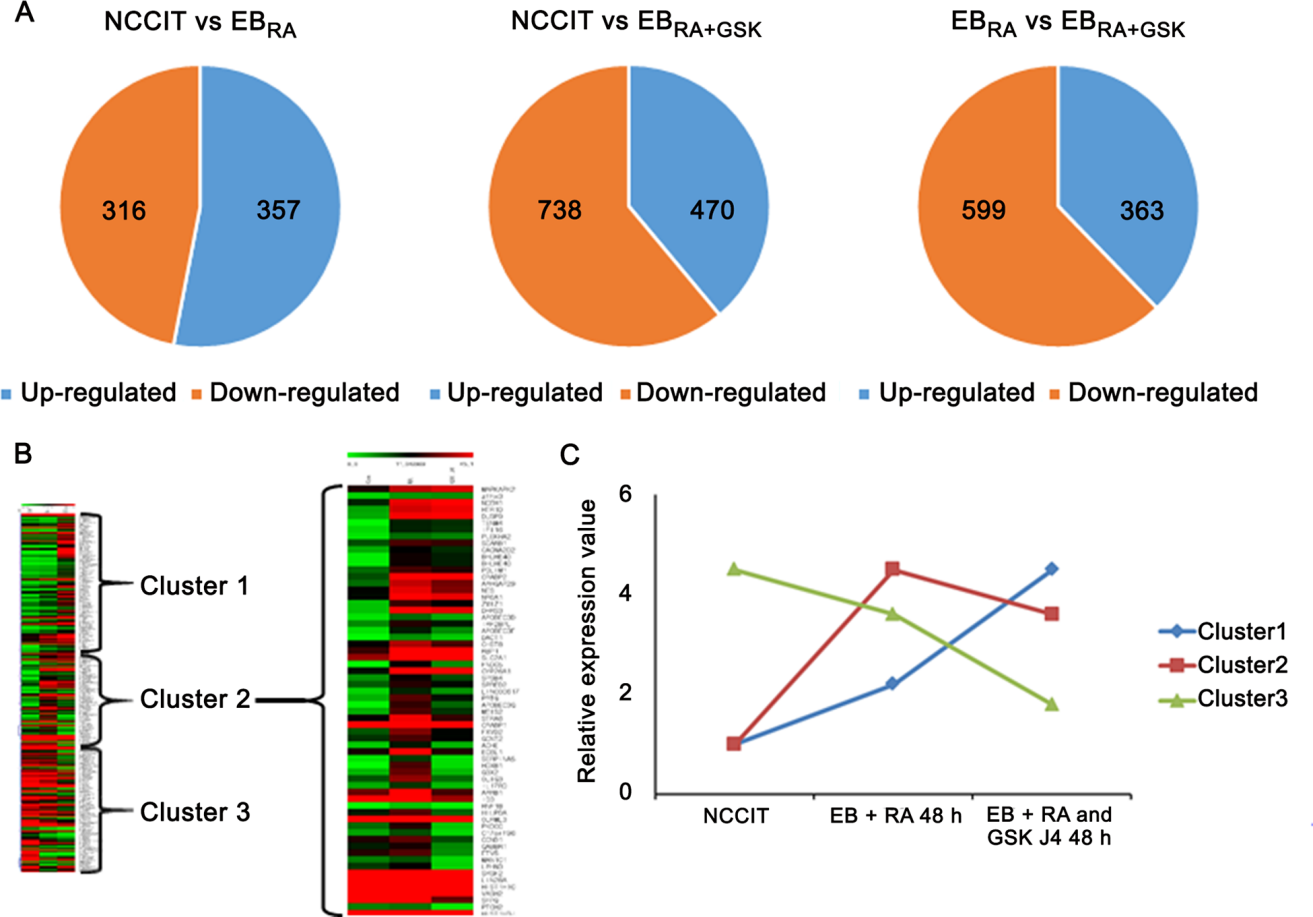
Transcriptomic profiling of RA-induced differentiation in NCCIT, EB_RA_ and EB_RA+GSK_. (A) Venn diagrams of the gene expression patterns in NCCIT, EB_RA_ and EB_RA+GSK_. The numbers represent the number of DEGs that were either up-regulated or down-regulated at the analyzed conditions. (B) Heat map representation of gene expression during RA-induced differentiation by RA and RA + GSK-J4 (green, low expression; red, high expression). Genes with specific expression patterns were then clustered into three groups (1, Up-Up; 2, Up-Down; 3, Down-Down). (C) Relative expression pattern graph of three gene clusters defined from the transcriptome profiling. Genes with specific expression patterns were clustered according to their relative expression values.

To identify developmental genes regulated by histone demethylation, the whole transcriptome of three samples was profiled using an Illumina HiSeq 2500: undifferentiated NCCIT, EB_RA_ and EB_RA+GSK_. Genes from RNA-seq with an absolute value of the log_2_ fold-change larger than two (log_2_ fold-change ≥ 2 and log_2_ fold-change ≤ -2) were considered to be differentially expressed genes (DEGs). A total of 673 genes were differentially expressed between NCCIT and EB_RA_, with 316 up-regulated and 357 down-regulated genes (Fig 3A). These DEGs were closely related to RA metabolism, cell differentiation and proliferation (Tables 2 and 3). GSK-J4 treatment increased the proportion of down-regulated genes in EB, and these DEGs were involved in gene transcription, cell differentiation and stress response (Tables 4 and 5). Comparing EB_RA_ with EB_RA+GSK_, a large number of genes (599 genes) were down-regulated, whereas a relatively small number of genes (363 genes) were activated. GSK-J4 treatment affected genes involved in stress response, gene transcription and cell specification (Tables 6 and 7). DEGs were also clustered into three groups according to the changes in their expression patterns (Fig 3B and 3C): up-regulation in both conditions (Cluster 1), staggered expression in response to RA and GSK-J4 treatments (Cluster 2), and exacerbated down-regulation (Cluster 3). Genes from Cluster 2 that showed deregulated transcriptional patterns after GSK-J4 treatment were used for further analyses.

**Table 2:** Top 10 up-regulated DEGs identified in EB_RA_ as compared to those of in NCCIT (*p* < 0.05).

Locus	Gene name	Entrez gene ID	Log_2_ fold-change	Putative function
XLOC_010497	HOXB1	3211	14.34569	Belongs to the homeobox family of genes; encode a highly conserved family of transcription factors that play an important role in morphogenesis in all multicellular organisms
XLOC_010326	HNF1B	6928	11.43815	Encodes a member of the homeodomain-containing superfamily of transcription factors; the gene has been shown to function in nephron development, and regulates development of the embryonic pancreas
XLOC_001950	CRABP2	1382	5.48966	Encodes a member of the RA binding protein family and lipocalin/cytosolic fatty-acid binding protein family; a cytosol-to-nuclear shuttling protein, which facilitates RA binding to its cognate receptor complex and transfer to the nucleus
XLOC_001452	FNDC5	252995	5.26276	Encodes a secreted protein that is released from muscle cells during exercise; may participate in the development of brown fat
XLOC_015495	APOBEC3G	60489	5.07141	A member of the cytidine deaminase gene family; may be RNA editing enzymes and have roles in growth or cell cycle control
XLOC_001298	DHRS3	9249	4.90678	A type of short-chain dehydrogenases/reductases (SDRs); catalyze the oxidation/reduction of a wide range of substrates, including retinoids and steroids
XLOC_004892	TENM4	26011	4.673	Involved in neural development
XLOC_022849	ZNF703	80139	4.61949	Acts as a transcriptional corepressor which does not bind directly to DNA and may regulate transcription through recruitment of histone deacetylases to gene promoters; regulates cell adhesion, migration and proliferation; may be required for segmental gene expression during hindbrain development
XLOC_010897	RBBP8	5932	4.32479	Encodes a ubiquitously expressed nuclear protein; complexes with transcriptional co-repressor CTBP; associated with BRCA1 and is thought to modulate the functions of BRCA1 in transcriptional regulation, DNA repair, and/or cell cycle checkpoint control
XLOC_006962	DACT1	51339	4.18477	Encodes a protein that belongs to the dapper family; interacts with, and positively regulates dishevelled-mediated signaling pathways during development

**Table 3:** Top 10 down-regulated DEGs identified in EB_RA_ as compared to those of in NCCIT (*p* < 0.05).

Locus	Gene name	Entrez gene ID	Log_2_ fold-change	Putative function
XLOC_017654	MXD4	10608	-5.91607	A member of the MAD gene family; tumor suppressors and contribute to the regulation of cell growth in differentiating tissues
XLOC_015744	ANKRD54	129138	-4.47717	Plays an important role in regulating intracellular signaling events associated with erythroid terminal differentiation
XLOC_010570	HEATR6	63897	-4.00643	Amplification-dependent oncogene
XLOC_021984	NRF1	4899	-3.48989	Encodes a protein that homodimerizes and functions as a transcription factor which activates the expression of some key metabolic genes regulating cellular growth and nuclear genes required for respiration, heme biosynthesis, and mitochondrial DNA transcription and replication; has also been associated with the regulation of neurite outgrowth
XLOC_016009	TMEM42	131616	-2.96713	Unknown
XLOC_000191	RHD	6007	-2.96435	Rh blood group, D antigen
XLOC_007413	OTX2	5015	-2.88437	Encodes a member of the bicoid subfamily of homeodomain-containing transcription factors; acts as a transcription factor and plays a role in brain, craniofacial, and sensory organ development
XLOC_011377	DAND5	199699	-2.8223	encodes a member of the BMP (bone morphogenic protein) antagonist family; may play a role in regulating organogenesis, body patterning, and tissue differentiation
XLOC_024410	WDR31	114987	-2.60329	Encodes a member of the WD repeat protein family; involved in a variety of cellular processes, including cell cycle progression, signal transduction, apoptosis, and gene regulation
XLOC_020332	THBS2	7058	-2.40784	Encoded by this gene belongs to the thrombospondin family; mediates cell-to-cell and cell-to-matrix interactions; shown to function as a potent inhibitor of tumor growth and angiogenesis

**Table 4:** Top 10 up-regulated DEGs identified in EB_RA+GSK_ as compared to those of in NCCIT (*p* < 0.05).

Locus	Gene name	Entrez gene ID	Log_2_ fold-change	Putative function
XLOC_012048	REXO1	9606	5.43387	Seems to have no detectable effect on transcription elongation in vitro
XLOC_008771	MT1E	4493	5.15957	Binds various heavy metals; transcriptionally regulated by both heavy metals and glucocorticoids
XLOC_016639	CELSR3	1951	5.1582	Belongs to the flamingo subfamily, which is included in the cadherin superfamily; may be involved in the regulation of contact-dependent neurite growth and may play a role in tumor formation
XLOC_010897	RBBP8	5932	4.98784	Encodes a ubiquitously expressed nuclear protein; complexes with transcriptional co-repressor CTBP; associated with BRCA1 and is thought to modulate the functions of BRCA1 in transcriptional regulation, DNA repair, and/or cell cycle checkpoint control
XLOC_001950	CRABP2	1382	4.88666	Encodes a member of the RA binding protein family and lipocalin/cytosolic fatty-acid binding protein family; a cytosol-to-nuclear shuttling protein, which facilitates RA binding to its cognate receptor complex and transfer to the nucleus
XLOC_023535	SCRIB	23513	4.68124	Encodes a protein that was identified as being similar to the Drosophila scribble protein; involved in tumor suppression pathways
XLOC_004892	TENM4	26011	4.59506	Involved in neural development
XLOC_008769	MT2A	4502	4.54637	Binds various heavy metals; transcriptionally regulated by both heavy metals and glucocorticoids
XLOC_017586	CPE	1363	4.28043	Encodes a carboxypeptidase that cleaves C-terminal amino acid residues and is involved in the biosynthesis of peptide hormones and neurotransmitters, including insulin
XLOC_001298	DHRS3	9249	4.00125	A type of short-chain dehydrogenases/reductases (SDRs); catalyze the oxidation/reduction of a wide range of substrates, including retinoids and steroids

**Table 5:** Top 10 down-regulated DEGs identified in EB_RA+GSK_ as compared to those of in NCCIT (*p* < 0.05).

Locus	Gene name	Entrez gene ID	Log_2_ fold-change	Putative function
XLOC_017654	MXD4	10608	-5.73052	A member of the MAD gene family; tumor suppressors and contribute to the regulation of cell growth in differentiating tissues
XLOC_000191	RHD	6007	-3.71237	Rh blood group, D antigen
XLOC_014222	TIGD1	200765	-3.61723	Belongs to the tigger subfamily of the pogo superfamily of DNA-mediated transposons in humans; the exact function of this gene is not known
XLOC_021984	NRF1	4899	-3.34274	Encodes a protein that homodimerizes and functions as a transcription factor which activates the expression of some key metabolic genes regulating cellular growth and nuclear genes required for respiration, heme biosynthesis, and mitochondrial DNA transcription and replication; has also been associated with the regulation of neurite outgrowth
XLOC_018012	SPOCK3	50859	-2.96754	encodes a member of a novel family of calcium-binding proteoglycan proteins that contain thyroglobulin type-1 and Kazal-like domains; may play a role in adult T-cell leukemia by inhibiting the activity of membrane-type matrix metalloproteinases
XLOC_010164	LINC00324	284029	-2.87243	An RNA gene; affiliated with the lncRNA class
XLOC_018419	VTRNA1-1	56664	-2.83112	An RNA gene; affiliated with the vault RNA class
XLOC_023491	HHLA1,OC90	10086	-2.63831	Unlikely to have a phospholipase A2 activity
XLOC_000258	LCK	3932	-2.62229	a member of the Src family of protein tyrosine kinases; a key signaling molecule in the selection and maturation of developing T-cells
XLOC_018056	SORBS2	8470	-2.53261	Adapter protein that plays a role in the assembling of signaling complexes, being a link between ABL kinases and actin cytoskeleton

**Table 6:** Top 10 up-regulated DEGs identified in EB_RA+GSK_ as compared to those of in EB_RA_ (*p* < 0.05).

Locus	Gene name	Entrez gene ID	Log_2_ fold-change	Putative function
XLOC_008771	MT1E	4493	5.65036	Binds various heavy metals; transcriptionally regulated by both heavy metals and glucocorticoids
XLOC_012048	REXO1	9606	5.18944	Seems to have no detectable effect on transcription elongation in vitro
XLOC_008769	MT2A	4502	5.15751	Binds various heavy metals; transcriptionally regulated by both heavy metals and glucocorticoids
XLOC_016639	CELSR3	1951	5.01109	Belongs to the flamingo subfamily, which is included in the cadherin superfamily; may be involved in the regulation of contact-dependent neurite growth and may play a role in tumor formation
XLOC_023535	SCRIB	23513	4.23912	Encodes a protein that was identified as being similar to the Drosophila scribble protein; involved in tumor suppression pathways
XLOC_023805	GADD45G	10912	3.59167	A member of a group of genes whose transcript levels are increased following stressful growth arrest conditions and treatment with DNA-damaging agents
XLOC_023770	ANXA1	301	3.59033	encodes a membrane-localized protein that binds phospholipids; inhibits phospholipase A2 and has anti-inflammatory activity
XLOC_002253	LEFTY2	7044	3.28842	encodes a member of the TGF-beta family of proteins; plays a role in left-right asymmetry determination of organ systems during development
XLOC_013015	MXD1	4084	3.04017	Encodes a member of the MYC/MAX/MAD network of basic helix-loop-helix leucine zipper transcription factors; mediates cellular proliferation, differentiation and apoptosis
XLOC_019332	HSPA1B	3304	3.02529	encodes a 70kDa heat shock protein which is a member of the heat shock protein 70 family; stabilizes existing proteins against aggregation and mediates the folding of newly translated proteins in the cytosol and in organelles; also involved in the ubiquitin-proteasome pathway

**Table 7:** Top 10 down-regulated DEGs identified in EB_RA+GSK_ as compared to those of in EB_RA_ (*p* < 0.05).

Locus	Gene name	Entrez gene ID	Log_2_ fold-change	Putative function
XLOC_014222	TIGD1	200765	-3.72327	Belongs to the tigger subfamily of the pogo superfamily of DNA-mediated transposons in humans; the exact function of this gene is not known
XLOC_014231	GBX2	2637	-3.04778	May act as a transcription factor for cell pluripotency and differentiation in the embryo
XLOC_012938	PKDCC	91461	-2.76946	Protein kinase which is required for longitudinal bone growth through regulation of chondrocyte differentiation
XLOC_004908	FZD4	8322	-2.72852	A member of the frizzled gene family; may play a role as a positive regulator of the Wingless type MMTV integration site signaling pathway
XLOC_007093	SERPINA5	5104	-2.64582	Encoded by this gene is a member of the serpin family of proteins; a glycoprotein that can inhibit several serine proteases, thus plays diverse roles in hemostasis and thrombosis in multiple organs
XLOC_001535	PTCH2	8643	-2.54595	Encodes a transmembrane receptor of the patched gene family; may function as a tumor suppressor in the hedgehog signaling pathway
XLOC_018072	LPHN3	23284	-2.51175	Encodes a member of the latrophilin subfamily of G-protein coupled receptors; may function in both cell adhesion and signal transduction
XLOC_001378	ID3	3399	-2.29516	A helix-loop-helix (HLH) protein that can form heterodimers with other HLH proteins; inhibits the DNA binding of any HLH protein with which it interacts
XLOC_020242	OLIG3	167826	-2.24445	May determine the distinct specification program of class A neurons in the dorsal part of the spinal cord and suppress specification of class B neurons
XLOC_010332	C17orf96	100170841	-2.17487	Has putative a nucleotide binding function

Selected DEGs affected by RA and GSK-J4 were submitted to the DAVID database for functional annotation. Genes up-regulated by GSK-J4 treatment were related to muscle development, ion transport, cellular homeostasis and anti-apoptosis (Table 8 and S1 Table), whereas those down-regulated by GSK-J4 were shown to participate in mesenchymal differentiation, embryonic organ morphogenesis, anterior/posterior pattern formation and cell adhesion (Table 9 and S2 Table). The GO annotation of genes in the EB_RA_ and EB_RA+GSK_ populations indicates that that inhibition of KDM6 demethylases deregulates some but not all differentiation processes. This suggests that KDM6 demethylase-independent developmental processes participate in early differentiation.

**Table 8:** Top 10 enriched processes for genes up-regulated in EB_RA+GSK_ compared to EB_RA_.

	GO term	Count	Fold enrichment
1	GO:0048634~regulation of muscle development	6	9.253078
2	GO:0016202~regulation of striated muscle tissue development	5	7.865116
3	GO:0006821~chloride transport	5	6.446817
4	GO:0030879~mammary gland development	5	6.242156
5	GO:0015698~inorganic anion transport	5	4.228557
5	GO:0032147~activation of protein kinase activity	6	4.139535
6	GO:0021700~developmental maturation	5	3.893622
7	GO:0060348~bone development	6	3.836642
8	GO:0046942~carboxylic acid transport	7	3.745293
9	GO:0015849~organic acid transport	7	3.719987
10	GO:0048634~regulation of muscle development	6	9.253078

**Table 9:** Top 10 enriched processes for genes down-regulated in EB_RA+GSK_ compared to EB_RA_.

	GO term	Count	Fold enrichment
1	GO:0048762~mesenchymal cell differentiation	5	7.801615
2	GO:0014031~mesenchymal cell development	5	7.801615
3	GO:0060485~mesenchyme development	5	7.651584
4	GO:0048704~embryonic skeletal system morphogenesis	5	6.980392
5	GO:0048754~branching morphogenesis of a tube	5	6.121267
5	GO:0019748~secondary metabolic process	6	6.043783
6	GO:0031349~positive regulation of defense response	5	5.450443
7	GO:0001763~morphogenesis of a branching structure	5	5.376789
8	GO:0048706~embryonic skeletal system development	5	5.167303
9	GO:0048562~embryonic organ morphogenesis	8	4.786555
10	GO:0048762~mesenchymal cell differentiation	5	7.801615

**Fig 4:**
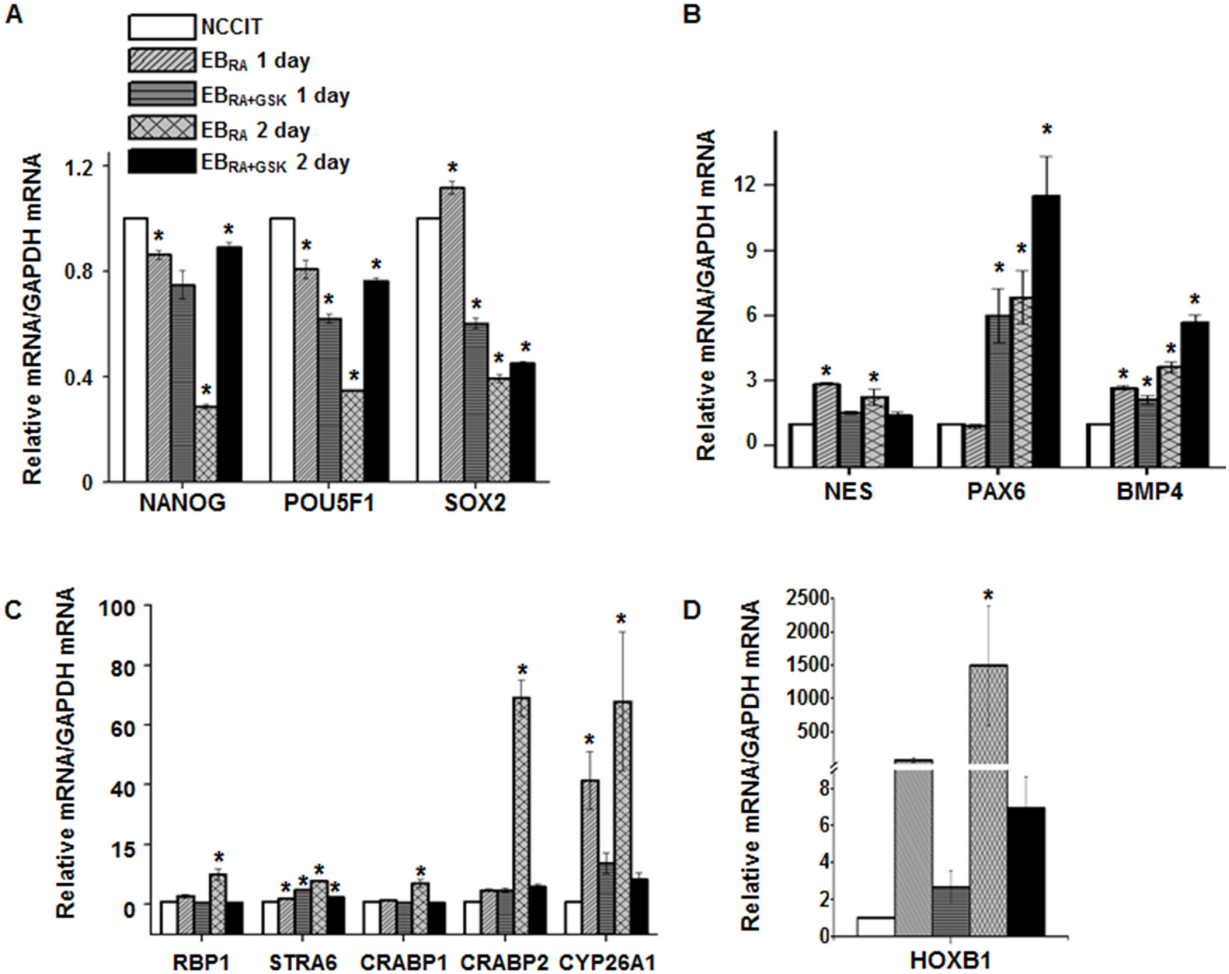
Validation of transcription patterns affected by GSK-J4 in cell differentiation. (A-D) mRNA expression levels of pluriopotency markers (A), neural markers (B) and RA-responsive genes (C-D) in RA-induced differentiation. qRT-PCR data were normalized to GAPDH expression. The values are presented as the means ± SE (n = 3). The asterisk represents the significant difference analyzed by a one-way ANOVA followed by Tukey’s HSD post hoc test (*: *P* < 0.05).

To validate our RNA-seq results, we assessed the expression of DEGs by qRT-PCR. RNA samples from RNA-seq were reverse transcribed into cDNA and subjected to qRT-PCR using a commercially available kit with specific primers. Because exogenous agents such as RA can induce neural differentiation of EC [41], we selected pluripotency markers (NANOG, SOX2, POU5F1), neural markers (NES, BMP4, PAX6) and other RA-related genes (RBP1, STRA6, CRABP1, CRABP2, CYP26A1 and HOXB1) as targets to assess RA-induced differentiation (Fig 4). The expression of pluripotency markers gradually decreased as RA-induced differentiation proceeded. Upon GSK-J4 treatment, the expression patterns of NANOG and POU5F1 were reversed, but that of SOX2 was not significantly affected (Fig 4A). Differentiation up-regulated the expression of three neural markers, but this change was less pronounced in NES than in the other two markers. Surprisingly, the inhibition of KDM6 demethylases enhanced the expression of PAX6 and BMP4, but the expression of NES decreased to a level similar to that observed in undifferentiated NCCIT cells (Fig 4B). RA-related genes showed similar expression patterns; their expression levels were increased by differentiation and reduced by GSK-J4 treatment. These results show that H3K27me3 demethylation regulates the transcription of many differentiation genes, whereas the regulation of some developmental genes is KDM6 demethylase-independent during the early stage of RA-induced differentiation.

### Global transcription and histone modification pattern characterization in early NCCIT differentiation

**Fig 5:**
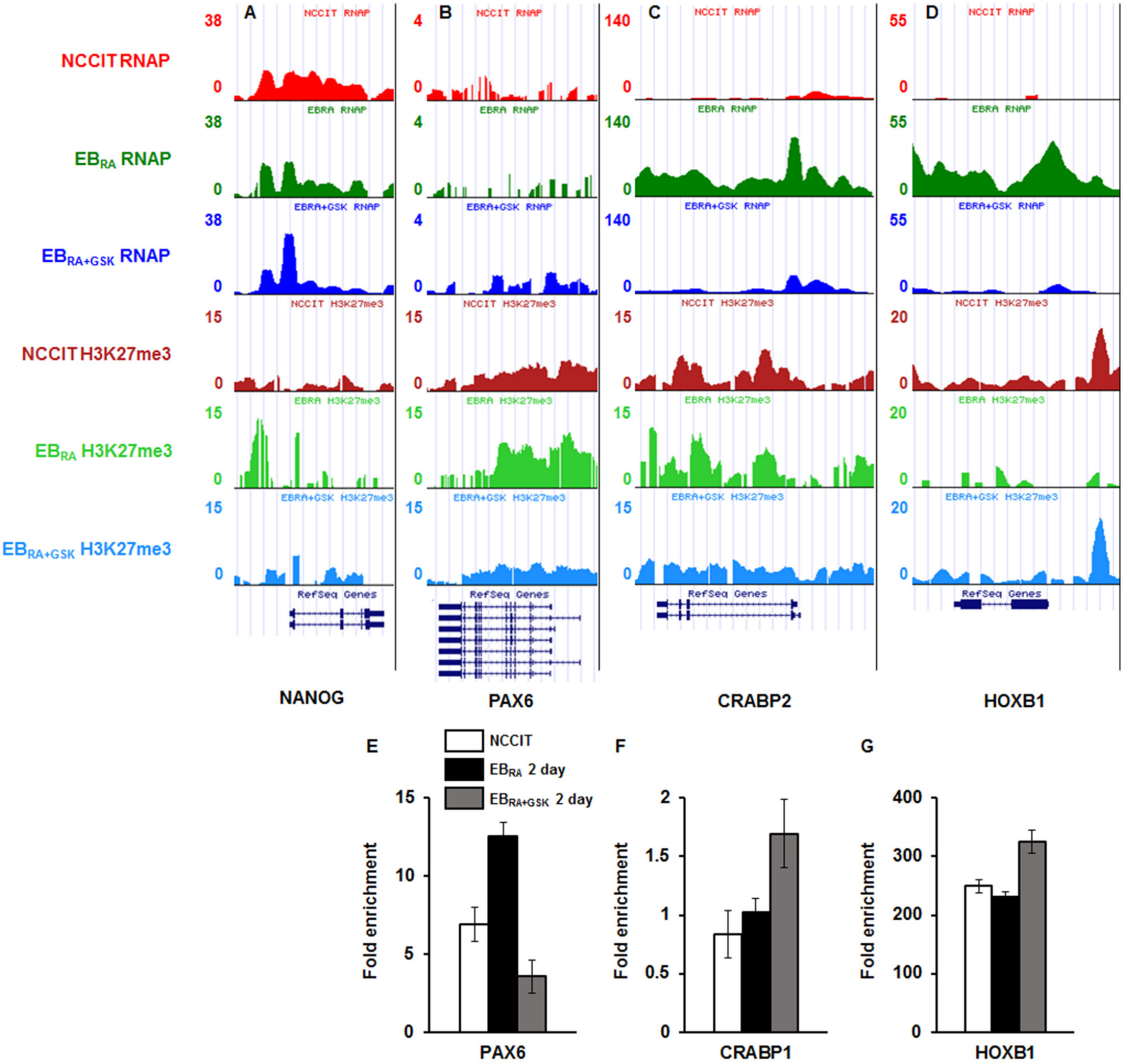
RNAP recruitment and H3K27me3 enrichment patterns in NCCIT, EB_RA_ and EB_RA+GSK_ in RA-induced differentiation. (A-D) USCS genome browser view of RNAPII binding and H3K27me3 enrichment in the promoters and the gene bodies of NANOG (A), PAX6 (B), CRABP2 (C) and HOXB1 (D). (E-G) ChIP-qPCR was performed on NCCIT cells using H3K27me3 antibody for PAX6 (E), CRABP1 (F) and HOXB1 (G) during RA-induced differentiation. qRT-PCR data were normalized to IgG expression. The values are presented as the means ± SE (n = 3).

To investigate the relationship between transcriptional activation and H3K27me3 repression in the early stage of RA-induced differentiation, we observed the RNAPII recruitment patterns as well as the H3K27me3 and H3K4me3 landscapes in NCCIT, EB_RA_ and EB_RA+GSK_ cells collected at day 2 (Fig 5 and S4 Fig). The sequenced raw reads were aligned to hg19, and enriched peaks were selected for analysis. Based on the transcriptome profiling data, we focused on RNAPII binding and H3K27me3 enrichment to the promoters of genes selected based on the RT-qPCR data: pluripotency markers (NANOG, SOX2, POU5F1), neural markers (NES, PAX6, BMP4) and RA-affected genes (RBP1, STRA6, CRABP1, CRABP2, CYP26A1, HOXB1).

The binding affinity of RNAPII to the promoters of pluripotency markers did not dramatically differ in EB_RA_ cells, and only a subtle increase could be observed in EB_RA+GSK_ cells. H3K27me3 enrichment to the upstream regions of pluripotency markers was increased upon RA induction and decreased as KDM6 demethylases were enzymatically inhibited (Fig 5A). The enrichment pattern of H3K27me3 in SOX2 was somewhat different; it was enriched at the start and the end of the SOX2 exon (S4 Fig). The H3K4me3 patterns were concordant with the aforementioned expression patterns observed by RNA-seq (S5 Fig). The RNAPII binding and H3K27me3 enrichment patterns showed that the transcription of stemness markers during differentiation are negatively mediated by H3K27me3 enrichment.

Neural marker promoters were enriched with RNAPII as differentiation was induced. This enrichment could also be observed in the gene-encoding regions of neural markers. Upon the GSK-J4 treatment of RA-treated NCCIT cells, the RNAPII enrichment in neural markers decreased at the promoter region and gene bodies of NES (S4 Fig). The RNAPII binding to the promoter regions and H3K4me3 recruitment near their transcription start sites (TSSs) of PAX6 and BMP4 were increased despite GSK-J4 treatment (Fig 5B, S4 and S5 Figs). These results are consistent with the aforementioned up-regulation in PAX6 and BMP4 expression in response to GSK-J4 treatment identified by RT-qPCR (Fig 4B). The H3K27me3 at the promoter regions of PAX6 and BMP4 increased as RA induced differentiation and decreased in response to GSK-J4 treatment. These results suggest that some developmental genes are activated in a KDM6 demethylase-independent manner.

The RNAPII binding and H3K4me3 recruitment patterns in five RA metabolism genes showed a similar pattern: increased binding in response to RA and reduced binding in response to GSK-J4 (Fig 5C, S4 and S5 Figs). In addition to RA metabolism, we selected HOXB1, one of the first targets regulated by RA signaling, to assess the role of H3K27me3 demethylases in the regulation of RA-mediated differentiation. Similar to RA metabolic genes, the RNAPII-binding and trimethylation of H3K4 near the HOXB1 promoter was enhanced in EB_RA_ cells and decreased in EB_RA+GSK_ cells (Fig 5D). The H3K27me3 enrichment patterns for RA-responding genes were similar: RA treatment induced the demethylation of H3K27me3, and GSK-J4 treatment impaired the enzymatic activity of KDM6 demethylases leading to H3K27me3 enrichment at the upstream region of the TSSs of RA-related genes. Thus, we surmised that the transcription initiation of RA-responsive metabolic and developmental genes is controlled by H3K27me3 demethylation. Collectively, the overall RNAPII recruitment and histone H3 methylation enrichment indicate that the transcription of key developmental genes is regulated in both KDM6 demethylase-dependent and KDM6 demethylase-independent manners.

In addition to ChIP-seq, ChIP-qPCR was used to further confirm histone modification enrichment. We attempted to assess the promoters of the aforementioned genes, but only three showed significant changes in their H3K27me3 demethylation patterns (Fig 5E–5G). In PAX6, the level of H3K27me3 increased in response to RA-induced differentiation and decreased in response to GSK-J4 treatment (Fig 5E). The increased H3K27me3 level during differentiation may reflect the histone demethylase-independent transcription of PAX6 [20], and the decreased level in response to GSK-J4 treatment partly explains the increased expression of PAX6 in EB_RA+GSK_ cells (Figs 4B and 5B). In RA-responsive genes, the inhibition of KDM6 demethylases by GSK-J4 led to increased H3K27me3 levels at their promoters (Fig 5F and 5G). Conclusively, both KDM6 demethylase-dependent and KDM6 demethylase-independent gene transcription co-exist during early differentiation.

### JMJD3 knockout and mRNA transcription pattern analysis of demethylase-dependent and demethylase-independent genes

**Fig 6:**
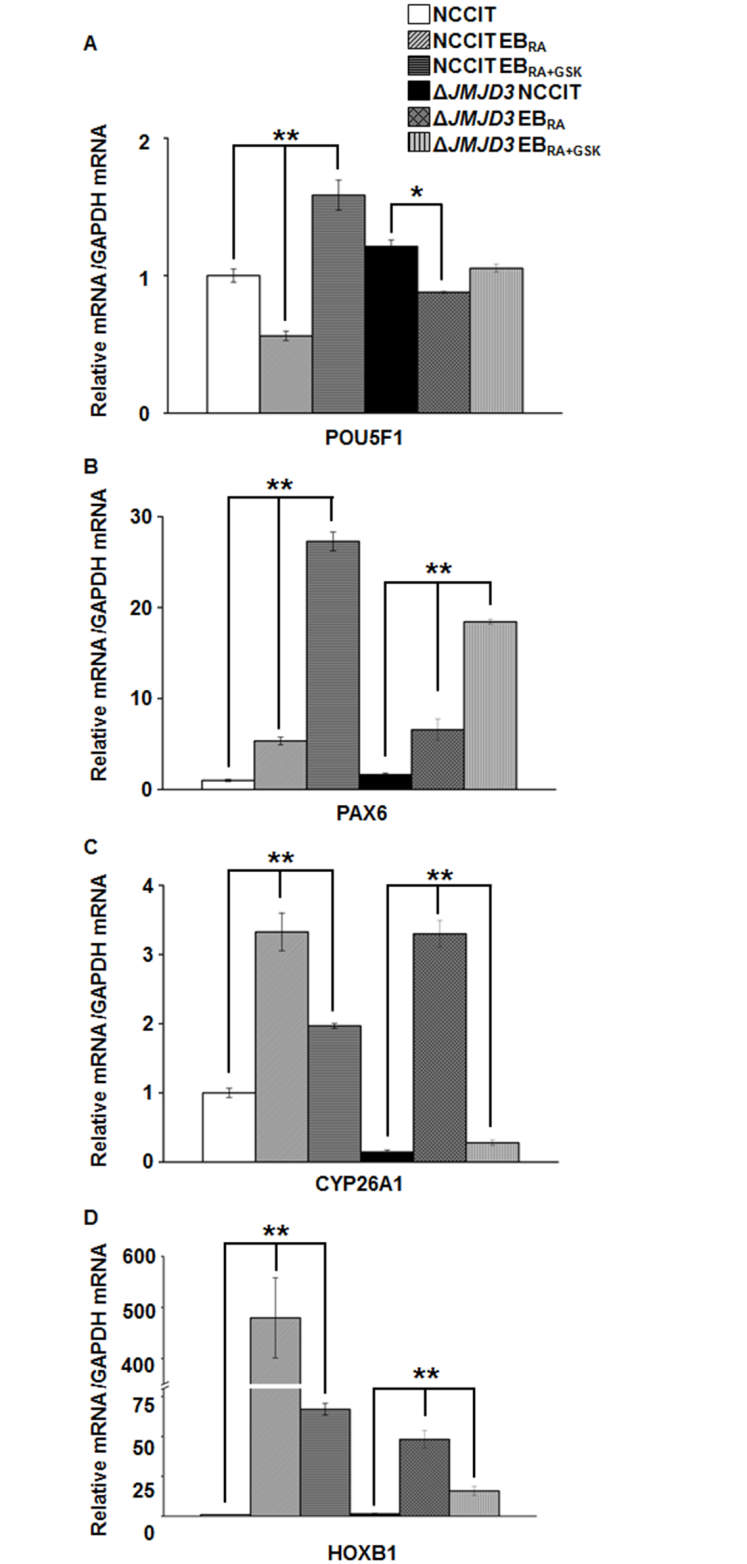
Transcription pattern changes in genes related to cell differentiation and KDM6 demethylase inhibition in *JMJD3* knockout NCCIT cells. (A-D) mRNA expression levels of a pluriopotency marker, POU5F1 (A), a neural marker, PAX6 (B) and the RA-responsive genes CYP26A1 and HOXB1 (C-D) during the RA-induced differentiation of *JMJD3* knockout cells. qRT-PCR data were normalized to GAPDH expression. The values are presented as the means ± SE (n = 3). The asterisk represents the significant difference analyzed with a one-way ANOVA followed by Tukey’s HSD post hoc test (*: *P* < 0.05; ** *P* < 0.01).

To further investigate the role of H3K27me3 demethylases during NCCIT differentiation, we constructed a JMJD3 knockout NCCIT cell line using CRISPR/Cas9 genome editing to examine the changes in the expression of selected genes. Initial attempts to abolish JMJD3 expression by deleting selected target sequences in JMJD3 exons 17, 21 or 22 produced insignificant changes in *JMJD3* mRNA expression (data not shown). Transfecting four target gRNA plasmids together into NCCIT cleaved a 1650-bp fragment ranging from exon 17 to 22, which significantly reduced *JMJD3* transcription in all cases of NCCIT differentiation and inhibition by GSK-J4 (S1B and S1C Fig). To assess the effect of JMJD3 knockout on the expression of differentiation-related and RA metabolism-related genes, the expression levels of the aforementioned genes were analyzed by qRT-PCR. None of the tested conditions significantly affected the mRNA expression levels of pluripotency markers, such as POU5F1, when JMJD3 was knocked down (Fig 6A), whereas NANOG and SOX2 mRNA were not re-expressed (S6A Fig). The expression patterns of neural differentiation markers were somewhat similar between wild-type and JMJD3 knockout cells under all conditions, differing only in the level of expression in response to GSK-J4 treatment, which did not significantly affect knockout cells. PAX6 and BMP4, which were predicted to be expressed in an H3K27me3 demethylase activity-independent manner, showed almost identical expression patterns under both conditions. The mRNA expression levels of RA metabolism genes were complex but decreased in most cases, indicating that H3K27me3 demethylation regulated the transcription of these genes. The significant decrease observed in the level of HOXB1 mRNA expression further confirmed the role of JMJD3 in gene transcription initiation. Similar differences in the mRNA levels may account for the compensation for *JMJD3* loss by UTX, analogous to the compensation of JMJD3 for *UTX* loss in mouse embryonic stem cells [20].

Collectively, the JMJD3 knockout experiments showed that KDM6 demethylase-dependent gene expression is largely regulated by JMJD3 and UTX plays only a minor role in this regulation. Furthermore, demethylase-independent gene expression can be cooperatively initiated by both JMJD3 and UTX.

## Discussion

Histone modifications play important roles in various biological processes, including differentiation. In this study, we used a selective JMJD/UTX inhibitor GSK-J4 that disables only the demethylase function of JMJD3 and UTX to study the role of H3K27me3 demethylation in RA-induced early neural differentiation. As an experimental model, we utilized NCCIT cells, whose differential traits are similar to those of human embryonic stem cells. Although these cells are considered to be stem-like cancer cells with potentially ambiguous phenotypes, they constitute a robust and simplified means to analyze the differentiation and development during human embryogenesis [42, 43]. Because RA-induced NCCIT differentiation has been widely in previous studies [44, 45], we selected this carcinoma cell line as a cellular model. A whole transcriptomic analysis and the genome-wide mapping of a transcriptional suppressive markers identified a complex relationship between transcription activation and changes in the epigenetic landscape due to the enzymatic activity of histone demethylases. We also found a mismatching correlation between transcription and H3K27 methylation at the regulatory regions of DEGs, suggesting the co-existence of KDM6 demethylase-dependent and KDM6 demethylase-independent gene regulation during the early differentiation of stem-like cancer cells.

The stimulation of NCCIT differentiation with RA induced the expression of gene subsets closely related to cell differentiation functions, such as energy metabolism, RA metabolic processing, cell specification, transcriptional activation, cell adhesion and proliferation (Tables 2, 3 and 8). The inhibition of KDM6 demethylases by GSK-J4 changed the types of genes expressed during RA-mediated differentiation (Tables 4–7 and 9), resulting in a delay of cell fate commitment. Genes that were up-regulated during differentiation showed decreased expression levels in response to GSK-J4 treatment, whereas genes related to stemness and transcriptional repression were readily expressed (Fig 4). Consistent with these transcriptome profiles, the H3K27me3 state of the regulatory elements of many JMJD3/UTX-dependent genes was subjected to demethylation for active transcription (Fig 5), although the efficiency of capturing H3K27me3 enrichment was lower than expected. JMJD3 knockout also reduced the down-regulation of pluripotency markers during differentiation (Fig 6A), further emphasizing the role of H3K27me3 and KDM6 demethylases in cell fate commitment. The histone methylation and transcriptional activation patterns of some developmental genes shown in this work are partly consistent with those described in previous reports of epigenetic changes that occur during differentiation: Regulatory regions of the pluripotency markers NANOG, SOX2 and POU5F1 are enriched with repressive markers during differentiation [46–50], but the presence of H3K27me3 is not considered to be an apparent blocker of this regulation, because the deletion of UTX does not compromise the transcription of these stemness markers [20]. In contrast to previous works, our study showed that the inhibition of H3K27me3 demethylase activity increased the mRNA expression of pluripotency markers (Fig 4A), suggesting the indirect regulation of pluripotency by H3K27me3 demethylation. Key differentiation effectors, such as NES, were regulated by H3K27me3 demethylation in this study (Fig 4B). This regulation is consistent with previous reports showing the necessity of JMJD3 in neural commitment and its direct regulation of gene transcription [4, 51]. The mRNA expression of some RA-processing enzymes also depended on demethylases (Figs 4C and 6C). CYP26A1 is a cytochrome P450 enzyme involved in the inactivation of RA that mediates the formation of the anterior-posterior axis [52] and establishes hindbrain patterning [53]. In mouse ES cells and F9 teratocarcinoma cells, CYP26A1 expression increases in response to RA treatment and decreases as RA is withdrawn, and an H3K27me3 demethylation pattern is observed [54, 55]. This demethylation may be due to the action of JMJD3 because the mRNA expression and the removal of H3K27me3 were affected by JMJD3/UTX inhibition. HOXB1, a homeobox gene that controls spatial specification and differentiation [56–58], contains an RA-responsive enhancer that enables its transcription in response to RA [59], to regulate differentiation into various tissue types. The role of HOXB1 in neural differentiation has been well-studied [60], and its regulation by H3K27me3 demethylase was also previously noted [5]. We noted that HOXB1 expression (Figs 4D and 6D) is responsive to RA and regulation via JMJD/UTX, and these findings are similar to those of previous studies. Collectively, our work signifies that the demethylation activity of JMJD3/UTX is important in RA-induced transcriptional initiation during development.

Unexpectedly, this study indicated complex patterns of histone modification contents for genes known to participate in RA-driven differentiation, and these patterns weakly correlated with the transcriptional activities of these genes (Figs 4, 5 and 6). In fact, the inhibition of JMJD3 and UTX did not affect the transcriptional patterns of several DEGs identified by RNA-seq (Tables 4–7). In this work, the regulatory sequences of PAX6 and BMP4 displayed confusing histone modification patterns during differentiation: their H3K27me3 levels increased as transcription was activated by RA treatment. Although the presence of H3K27me3 may reflect its inhibitory role in transcription, previous studies showed that the demethylation of H3K27me3 does not significantly affect the transcriptional efficiency of PAX6 and rather enhances its expression level to some extent [20], similar to the findings reported herein. This demethylase-independent transcriptional regulatory role may be attributed to the functions of JMJD3 and UTX, which mediate an interaction between the T-box transcription factor and the SWI/SNF complex [22]. The T-box family proteins regulate the transcription of genes involved in various cellular development processes [61], and these T-box factors interact with H3K27me3 demethylases to activate genes in developing and static cells [62]. JMJD3 and UTX mediate the interaction between the T-box transcription factor T-bet and the Brg1-containing SWI/SNF remodeling complex, further enhancing their gene regulatory roles in differentiated cells [22]. Because this study focuses on early differentiation, gene activation by JMJD3/UTX via the T-bet and SWI/SNF complex may already occur by the 48-h mark. Alternatively, JMJD3/UTX-independent H3K27me3 demethylation may underlie the inconsistency between transcriptional initiation and the removal of histone-suppressive markers. Although UTX and JMJD3 are essential for the demethylation of key development effectors, such as HOX family proteins, cells lacking one or both KDM6 demethylases show minor changes in H3K27me3 demethylation patterns and may survive to differentiate [23]. The exact mechanisms responsible for this phenomenon have yet to be elucidated; nonetheless, KDM6-independent H3K27me3 demethylation may occur during early differentiation. Genes whose promoters previously demonstrated a loss of H3K27me3 in UTX-and-JMJD3-deleted ES cells [23] also showed similar patterns in our data, although the peak is weak (data not shown), indicating that both H3K27me3-specific demethylase-dependent and demethylase-independent histone demethylation can co-exist during cell commitment.

Histone modifications play critical roles in biological processes, but their mechanisms are not fully understood. Additionally, the histone code is not consistent with the phenotypes and requires further interpretation. Differentiating cells undergo rapid changes in transcription and translation, requiring not only expeditious chromatin modifications but also the fine-tuning of transcriptional initiation. As reported by Heinemann’s group [63], GSK-J4 may have unintentionally inhibited other KDM family members, which may have affected the results of this study. Nevertheless, we showed the genome-wide scope of H3K27me3-related changes that occurred during the early differentiation of a carcinoma cell line and the consequent changes in its transcriptome, providing demethylase-dependent and demethylase-independent transcription profiles. Further analyses of chromatin structures, other histone modification contents and binding of transcription factors to regulatory sites where the conventional idea of repression by H3K27 methylation is inconsistent with observed phenotypes may provide insight into the elaborate epigenetic regulatory system of differentiating cells.

## Supporting Information

S1 Fig Construction of *JMJD3* knockout NCCIT cell line using the CRISPR/Cas9 system. (A) Schematic illustration of constructing *JMJD3* knockout NCCIT using the CRISPR/Cas9 system. Total four sequence fragments were targeted using separate gRNAs that were expressed in the transfected NCCIT. (B) mRNA expression of *JMJD3* and *UTX* in WT NCCIT and *JMJD3* knockout cell. (C) mRNA expression patterns of *JMJD3* during RA-induced differentiation and GSK-J4 inhibition. qRT-PCR were normalized for GAPDH. The values are presented as the means ± SE (n = 3). The asterisk represents the significant difference analyzed by one-way ANOVA followed by Tukey’s HSD post hoc test (*: *P* < 0.05; ** *P* < 0.01).
(TIF)

S2 Fig The effect of GSK-J4 in the cell morphological changes during RA-induced NCCIT differentiation. (TIF)

S3 Fig Inhibition of JMJD3/UTX in EB by GSK-J4 during RA-induced NCCIT differentiation. (TIF)

S4 Fig The effect of GSK-J4 in RNAPII recruitment and H3K27me3 enrichment patterns of selected genes differentially expressed during RA-induced NCCIT differentiation. (TIF)

S5 Fig The effect of GSK-J4 in H3K4me3 enrichment patterns of selected genes differentially expressed during RA-induced NCCIT differentiation. (TIF)

S6 Fig Gene transcription pattern analyses of selected genes in *JMJD3* knockout NCCIT during cell differentiation and KDM6 demethylase inhibition. (A-D) mRNA expression levels of pluriopotency markers (A), neural markers (B) and RA-responsive genes (C-D) in RA-induced differentiation of *JMJD3* knockout cells. qRT-PCR were normalized for GAPDH. The values are presented as the means ± SE (n = 3).
(TIF)

S1 Table Enriched processes for genes up-regulated in EB_RA+GSK_ compared to EB_RA_. (DOCX)

S2 Table Enriched processes for genes down-regulated in EB_RA+GSK_ compared to EB_RA_. (DOCX)
