# Supplementary figures and images for: Transcriptomic Profiling and H3K27me3 Distribution Reveal Both Demethylase-Dependent and Independent Regulation of Developmental Gene Transcription in Cell Differentiation
Source: PLoS One. 2015 Aug 11;10(8):e0135276. doi: 10.1371/journal.pone.0135276 (PMC4532468; doi:10.1371/journal.pone.0135276)

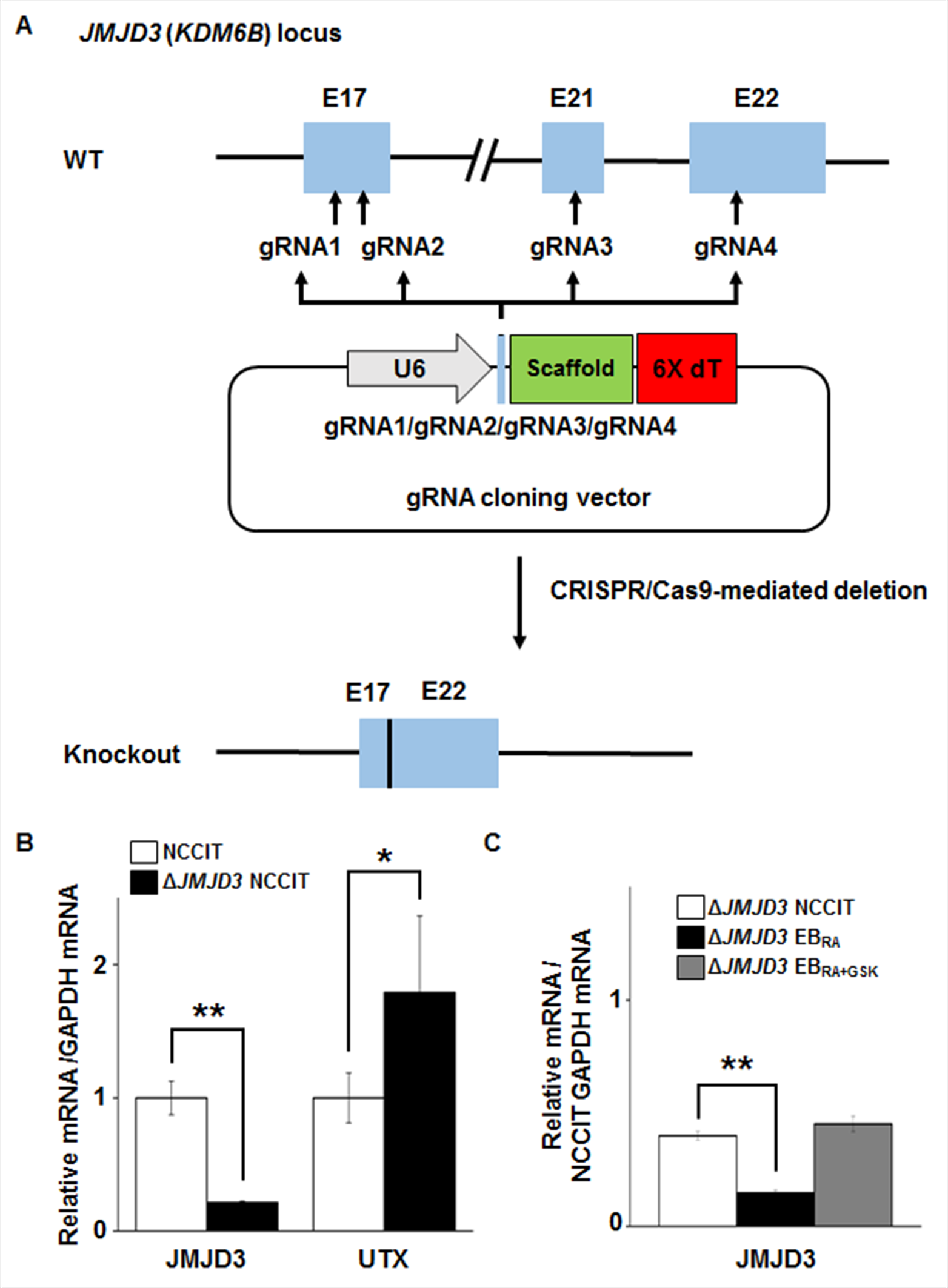

Supplement: S1 Fig — (A) Schematic illustration of constructing JMJD3 knockout NCCIT using the CRISPR/Cas9 system. Total four sequence fragments were targeted using separate gRNAs that were expressed in the transfected NCCIT. (B) mRNA expression of JMJD3 and UTX in WT NCCIT and JMJD3 knockout cell. (C) mRNA expression patterns of JMJD3 during RA-induced differentiation and GSK-J4 inhibition. qRT-PCR were normalized for GAPDH. The values are presented as the means ± SE (n = 3). The asterisk represents the significant difference analyzed by one-way ANOVA followed by Tukey’s HSD post hoc test (*: P < 0.05; ** P < 0.01). (TIF) [file pone.0135276.s001.tif]

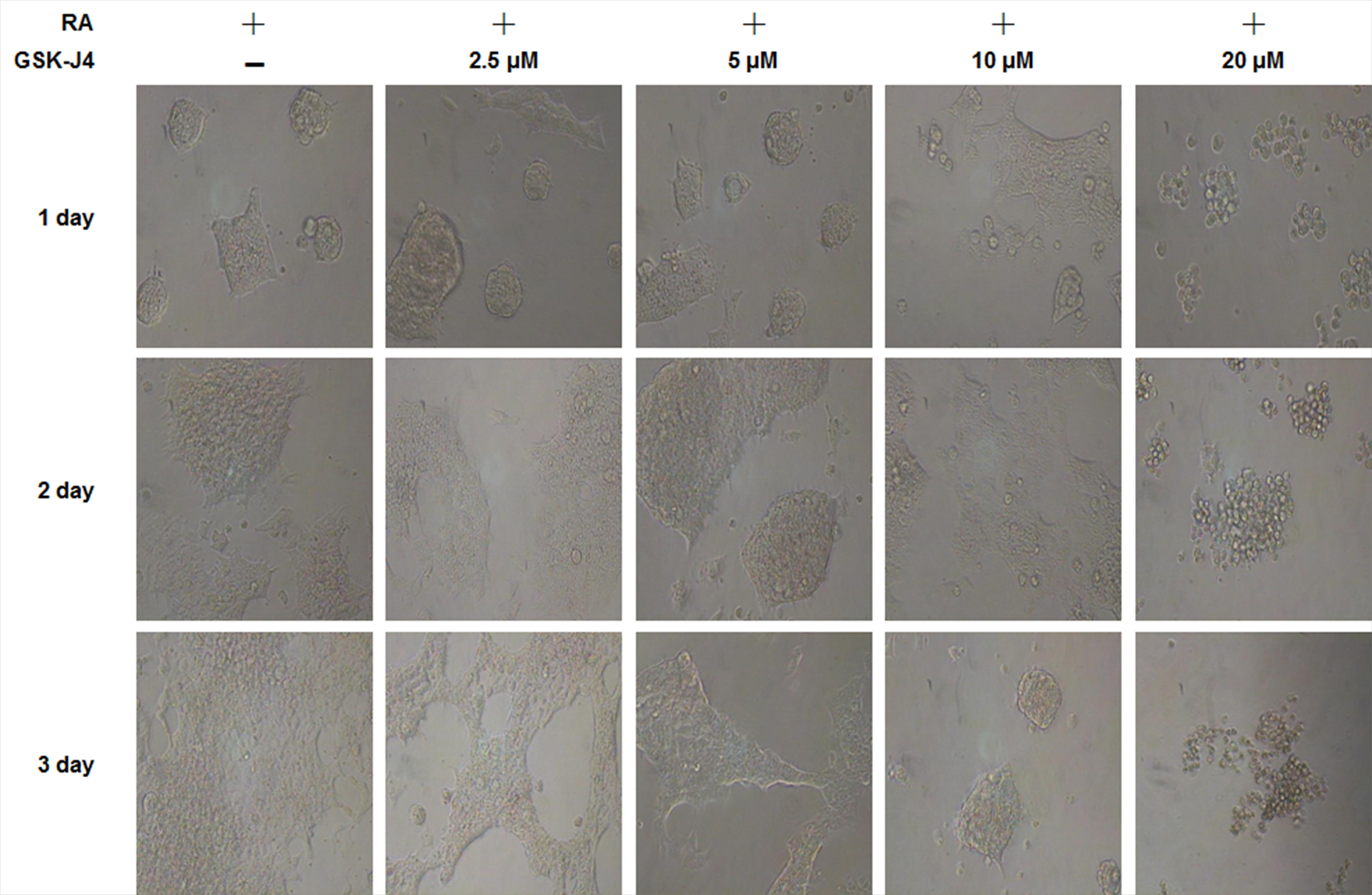

Supplement: S2 Fig — (TIF) [file pone.0135276.s002.tif]

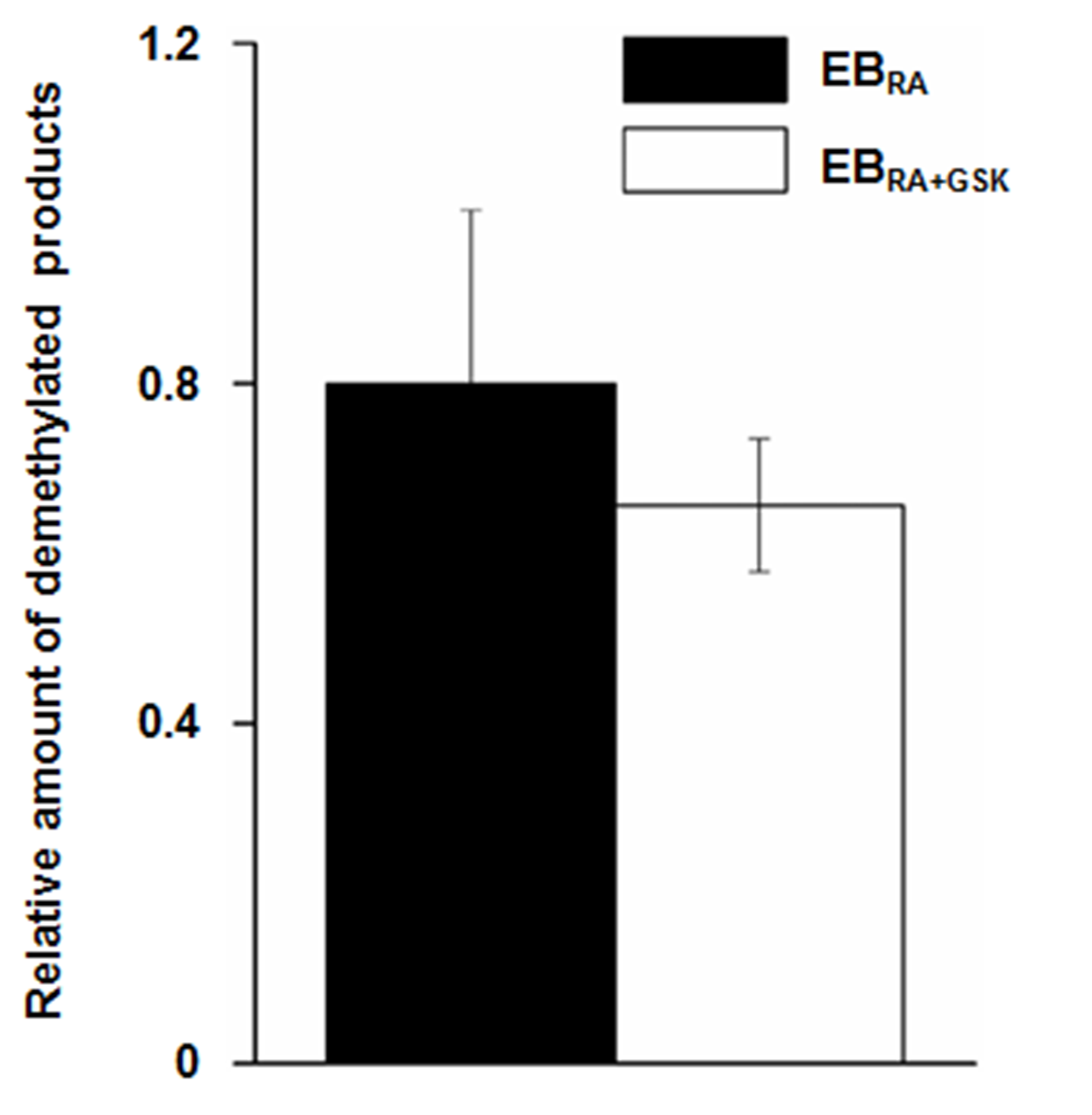

Supplement: S3 Fig — (TIF) [file pone.0135276.s003.tif]

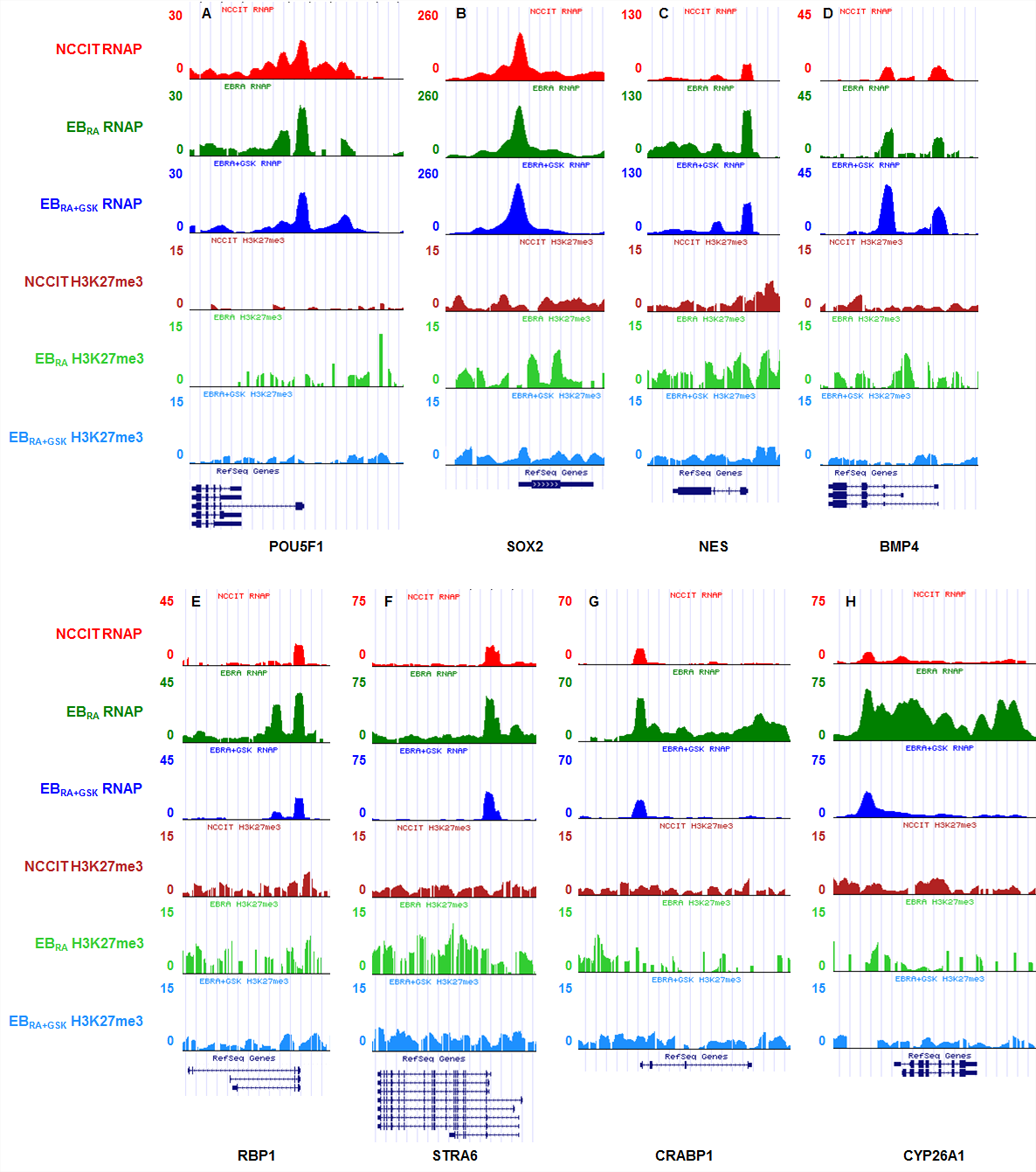

Supplement: S4 Fig — (TIF) [file pone.0135276.s004.tif]

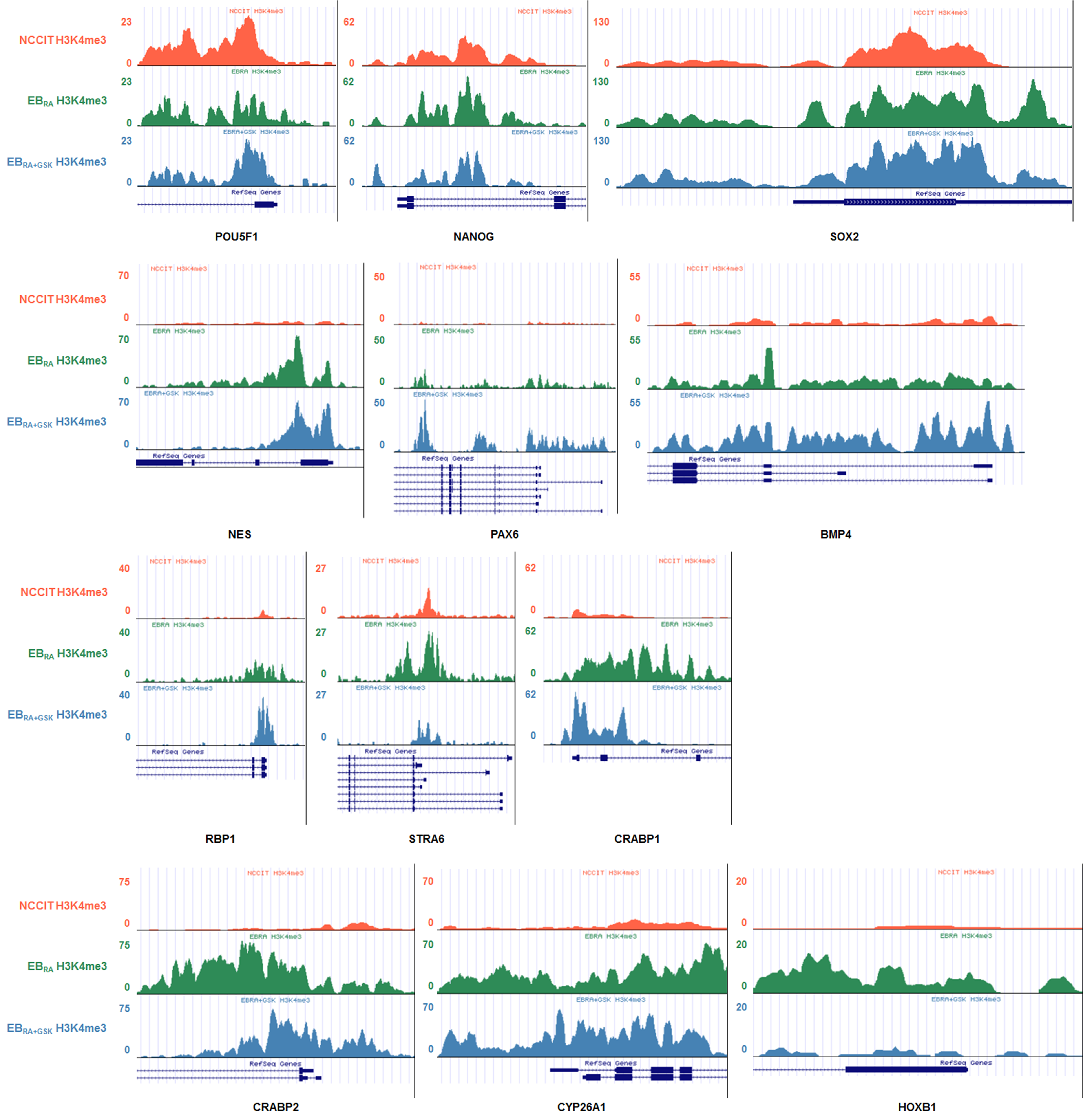

Supplement: S5 Fig — (TIF) [file pone.0135276.s005.tif]

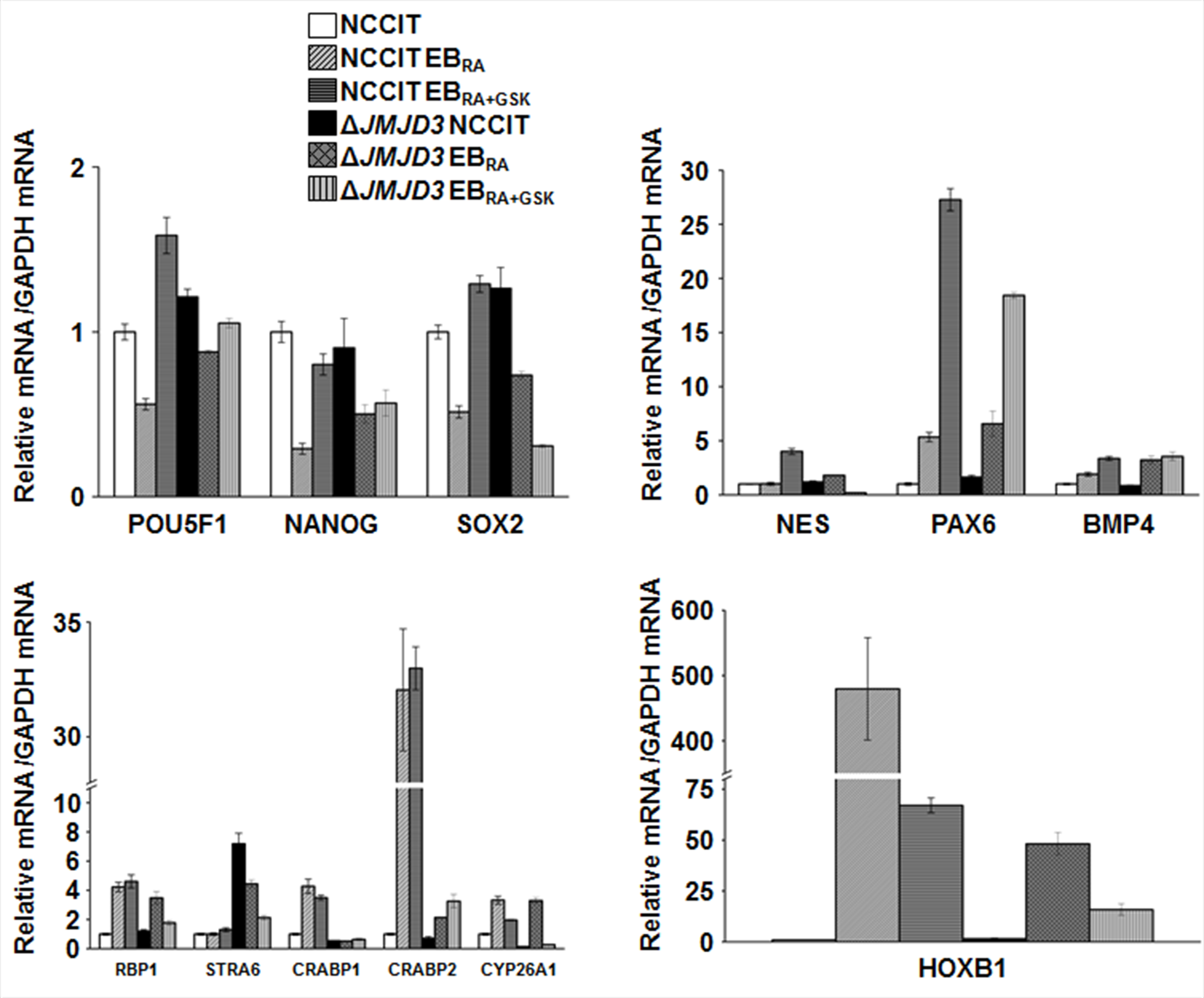

Supplement: S6 Fig — (A-D) mRNA expression levels of pluriopotency markers (A), neural markers (B) and RA-responsive genes (C-D) in RA-induced differentiation of JMJD3 knockout cells. qRT-PCR were normalized for GAPDH. The values are presented as the means ± SE (n = 3). (TIF) [file pone.0135276.s006.tif]
