# Supplementary material for: Transcriptomic Profiling and H3K27me3 Distribution Reveal Both Demethylase-Dependent and Independent Regulation of Developmental Gene Transcription in Cell Differentiation
Source: PLoS One. 2015 Aug 11;10(8):e0135276. doi: 10.1371/journal.pone.0135276 (PMC4532468; doi:10.1371/journal.pone.0135276)
Supplement: S1 Table — (DOCX) [file pone.0135276.s007.docx]

**S1 Table. Enriched processes for genes up-regulated in EB_RA+GSK_ compared to EB_RA_.**

|  | GO term | Count | Fold enrichment |
| --- | --- | --- | --- |
| 1 | GO:0048634~regulation of muscle development | 6 | 9.253077975 |
| 2 | GO:0016202~regulation of striated muscle tissue development | 5 | 7.865116279 |
| 3 | GO:0006821~chloride transport | 5 | 6.446816622 |
| 4 | GO:0030879~mammary gland development | 5 | 6.242155777 |
| 5 | GO:0015698~inorganic anion transport | 5 | 4.228557139 |
| 6 | GO:0032147~activation of protein kinase activity | 6 | 4.139534884 |
| 7 | GO:0021700~developmental maturation | 5 | 3.89362192 |
| 8 | GO:0060348~bone development | 6 | 3.836642087 |
| 9 | GO:0046942~carboxylic acid transport | 7 | 3.745293466 |
| 10 | GO:0015849~organic acid transport | 7 | 3.719987429 |
| 11 | GO:0042445~hormone metabolic process | 5 | 3.709960509 |
| 12 | GO:0010817~regulation of hormone levels | 7 | 3.646080394 |
| 13 | GO:0032583~regulation of gene-specific transcription | 6 | 3.521693856 |
| 14 | GO:0048732~gland development | 6 | 3.495607235 |
| 15 | GO:0001503~ossification | 5 | 3.419615774 |
| 16 | GO:0006816~calcium ion transport | 6 | 3.323288569 |
| 17 | GO:0014706~striated muscle tissue development | 5 | 3.304670705 |
| 18 | GO:0040008~regulation of growth | 14 | 3.229079997 |
| 19 | GO:0060537~muscle tissue development | 5 | 3.146046512 |
| 20 | GO:0030900~forebrain development | 6 | 3.104651163 |
| 21 | GO:0045137~development of primary sexual characteristics | 5 | 3.096502472 |
| 22 | GO:0035239~tube morphogenesis | 5 | 3.096502472 |
| 23 | GO:0048729~tissue morphogenesis | 7 | 3.058656331 |
| 24 | GO:0006916~anti-apoptosis | 8 | 3.054414089 |
| 25 | GO:0006814~sodium ion transport | 5 | 3.025044723 |
| 26 | GO:0007156~homophilic cell adhesion | 5 | 3.001952778 |
| 27 | GO:0051240~positive regulation of multicellular organismal process | 9 | 2.90106748 |
| 28 | GO:0051493~regulation of cytoskeleton organization | 5 | 2.891586867 |
| 29 | GO:0010647~positive regulation of cell communication | 12 | 2.868735421 |
| 30 | GO:0009991~response to extracellular stimulus | 8 | 2.860042283 |
| 31 | GO:0007409~axonogenesis | 7 | 2.852632847 |
| 32 | GO:0006875~cellular metal ion homeostasis | 7 | 2.8089701 |
| 33 | GO:0003002~regionalization | 7 | 2.794711368 |
| 34 | GO:0030003~cellular cation homeostasis | 9 | 2.786852225 |
| 35 | GO:0060429~epithelium development | 8 | 2.771847147 |
| 36 | GO:0008202~steroid metabolic process | 7 | 2.725535344 |
| 37 | GO:0055065~metal ion homeostasis | 7 | 2.685649461 |
| 38 | GO:0060284~regulation of cell development | 7 | 2.685649461 |
| 39 | GO:0015674~di-, tri-valent inorganic cation transport | 6 | 2.681289641 |
| 40 | GO:0009967~positive regulation of signal transduction | 10 | 2.666141112 |
| 41 | GO:0007389~pattern specification process | 9 | 2.651162791 |
| 42 | GO:0048667~cell morphogenesis involved in neuron differentiation | 7 | 2.634249471 |
| 43 | GO:0007267~cell-cell signaling | 20 | 2.621705426 |
| 44 | GO:0048812~neuron projection morphogenesis | 7 | 2.584779998 |
| 45 | GO:0000904~cell morphogenesis involved in differentiation | 8 | 2.578726649 |
| 46 | GO:0006874~cellular calcium ion homeostasis | 6 | 2.578726649 |
| 47 | GO:0055074~calcium ion homeostasis | 6 | 2.510143493 |
| 48 | GO:0035295~tube development | 7 | 2.502536998 |
| 49 | GO:0055080~cation homeostasis | 9 | 2.475036591 |
| 50 | GO:0045860~positive regulation of protein kinase activity | 7 | 2.468870581 |
| 51 | GO:0001501~skeletal system development | 10 | 2.465553692 |
| 52 | GO:0031175~neuron projection development | 8 | 2.457848837 |
| 53 | GO:0043066~negative regulation of apoptosis | 11 | 2.443962686 |
| 54 | GO:0001558~regulation of cell growth | 6 | 2.432510189 |
| 55 | GO:0030005~cellular di-, tri-valent inorganic cation homeostasis | 7 | 2.425366253 |
| 56 | GO:0006954~inflammatory response | 10 | 2.420035778 |
| 57 | GO:0043069~negative regulation of programmed cell death | 11 | 2.409924208 |
| 58 | GO:0060548~negative regulation of cell death | 11 | 2.403229974 |
| 59 | GO:0033674~positive regulation of kinase activity | 7 | 2.383368569 |
| 60 | GO:0007610~behavior | 14 | 2.347795904 |
| 61 | GO:0048666~neuron development | 10 | 2.320093298 |
| 62 | GO:0006873~cellular ion homeostasis | 11 | 2.313269494 |
| 63 | GO:0050801~ion homeostasis | 12 | 2.307613578 |
| 64 | GO:0055066~di-, tri-valent inorganic cation homeostasis | 7 | 2.303590542 |
| 65 | GO:0042127~regulation of cell proliferation | 23 | 2.298572737 |
| 66 | GO:0007626~locomotory behavior | 8 | 2.296384315 |
| 67 | GO:0051347~positive regulation of transferase activity | 7 | 2.293992248 |
| 68 | GO:0055082~cellular chemical homeostasis | 11 | 2.276744186 |
| 69 | GO:0048858~cell projection morphogenesis | 7 | 2.24717608 |
| 70 | GO:0008285~negative regulation of cell proliferation | 10 | 2.17870257 |
| 71 | GO:0046903~secretion | 8 | 2.097364341 |
| 72 | GO:0030001~metal ion transport | 12 | 2.029707427 |
| 73 | GO:0019725~cellular homeostasis | 12 | 2.025351832 |
| 74 | GO:0048878~chemical homeostasis | 13 | 1.99700218 |
| 75 | GO:0006812~cation transport | 14 | 1.991168678 |
| 76 | GO:0000902~cell morphogenesis | 9 | 1.988372093 |
| 77 | GO:0006928~cell motion | 12 | 1.986976744 |
| 78 | GO:0030182~neuron differentiation | 11 | 1.975257513 |
| 79 | GO:0006811~ion transport | 19 | 1.945796996 |
| 80 | GO:0009611~response to wounding | 13 | 1.929179465 |
| 81 | GO:0030030~cell projection organization | 9 | 1.923533873 |
| 82 | GO:0042981~regulation of apoptosis | 19 | 1.858671757 |
| 83 | GO:0043067~regulation of programmed cell death | 19 | 1.84035972 |
| 84 | GO:0010941~regulation of cell death | 19 | 1.83358539 |
| 85 | GO:0043085~positive regulation of catalytic activity | 12 | 1.815026834 |
| 86 | GO:0042592~homeostatic process | 15 | 1.570928684 |
