# Supplementary material for: Transcriptomic Profiling and H3K27me3 Distribution Reveal Both Demethylase-Dependent and Independent Regulation of Developmental Gene Transcription in Cell Differentiation
Source: PLoS One. 2015 Aug 11;10(8):e0135276. doi: 10.1371/journal.pone.0135276 (PMC4532468; doi:10.1371/journal.pone.0135276)
Supplement: S2 Table — (DOCX) [file pone.0135276.s008.docx]

**S2 Table. Enriched processes for genes down-regulated in EB_RA+GSK_ compared to EB_RA_.**

|  | GO term | Count | Fold Enrichment |
| --- | --- | --- | --- |
| 1 | GO:0048762~mesenchymal cell differentiation | 5 | 7.801614764 |
| 2 | GO:0014031~mesenchymal cell development | 5 | 7.801614764 |
| 3 | GO:0060485~mesenchyme development | 5 | 7.65158371 |
| 4 | GO:0048704~embryonic skeletal system morphogenesis | 5 | 6.980392157 |
| 5 | GO:0048754~branching morphogenesis of a tube | 5 | 6.121266968 |
| 6 | GO:0019748~secondary metabolic process | 6 | 6.043782576 |
| 7 | GO:0031349~positive regulation of defense response | 5 | 5.450443191 |
| 8 | GO:0001763~morphogenesis of a branching structure | 5 | 5.376788553 |
| 9 | GO:0048706~embryonic skeletal system development | 5 | 5.167303285 |
| 10 | GO:0048562~embryonic organ morphogenesis | 8 | 4.786554622 |
| 11 | GO:0002237~response to molecule of bacterial origin | 5 | 4.626538988 |
| 12 | GO:0045087~innate immune response | 8 | 4.61312873 |
| 13 | GO:0009206~purine ribonucleoside triphosphate biosynthetic process | 5 | 4.06002401 |
| 14 | GO:0009201~ribonucleoside triphosphate biosynthetic process | 5 | 4.019013666 |
| 15 | GO:0009145~purine nucleoside triphosphate biosynthetic process | 5 | 4.019013666 |
| 16 | GO:0043269~regulation of ion transport | 5 | 4.019013666 |
| 17 | GO:0009952~anterior/posterior pattern formation | 7 | 3.978823529 |
| 18 | GO:0009142~nucleoside triphosphate biosynthetic process | 5 | 3.900807382 |
| 19 | GO:0048568~embryonic organ development | 8 | 3.70123119 |
| 20 | GO:0009141~nucleoside triphosphate metabolic process | 6 | 3.644723844 |
| 21 | GO:0048705~skeletal system morphogenesis | 5 | 3.552521008 |
| 22 | GO:0009152~purine ribonucleotide biosynthetic process | 5 | 3.400703871 |
| 23 | GO:0009205~purine ribonucleoside triphosphate metabolic process | 5 | 3.400703871 |
| 24 | GO:0009199~ribonucleoside triphosphate metabolic process | 5 | 3.371884347 |
| 25 | GO:0009144~purine nucleoside triphosphate metabolic process | 5 | 3.261330762 |
| 26 | GO:0006164~purine nucleotide biosynthetic process | 6 | 3.226073132 |
| 27 | GO:0009260~ribonucleotide biosynthetic process | 5 | 3.208728653 |
| 28 | GO:0035239~tube morphogenesis | 5 | 3.132931913 |
| 29 | GO:0002252~immune effector process | 5 | 2.969271291 |
| 30 | GO:0030855~epithelial cell differentiation | 5 | 2.904250751 |
| 31 | GO:0009617~response to bacterium | 7 | 2.886193234 |
| 32 | GO:0009150~purine ribonucleotide metabolic process | 5 | 2.883205456 |
| 33 | GO:0006575~cellular amino acid derivative metabolic process | 6 | 2.876257973 |
| 34 | GO:0003002~regionalization | 7 | 2.827590325 |
| 35 | GO:0048584~positive regulation of response to stimulus | 8 | 2.697507478 |
| 36 | GO:0048729~tissue morphogenesis | 6 | 2.65254902 |
| 37 | GO:0045321~leukocyte activation | 8 | 2.630627127 |
| 38 | GO:0008544~epidermis development | 6 | 2.59488491 |
| 39 | GO:0006163~purine nucleotide metabolic process | 6 | 2.566982922 |
| 40 | GO:0009165~nucleotide biosynthetic process | 6 | 2.566982922 |
| 41 | GO:0035295~tube development | 7 | 2.53197861 |
| 42 | GO:0034654~nucleobase, nucleoside, nucleotide and nucleic acid biosynthetic process | 6 | 2.473879915 |
| 43 | GO:0034404~nucleobase, nucleoside and nucleotide biosynthetic process | 6 | 2.473879915 |
| 44 | GO:0060429~epithelium development | 7 | 2.453899974 |
| 45 | GO:0007423~sensory organ development | 7 | 2.432468533 |
| 46 | GO:0007389~pattern specification process | 8 | 2.384313725 |
| 47 | GO:0008283~cell proliferation | 13 | 2.37269293 |
| 48 | GO:0048598~embryonic morphogenesis | 9 | 2.332860701 |
| 49 | GO:0006955~immune response | 20 | 2.306564365 |
| 50 | GO:0006811~ion transport | 22 | 2.279534314 |
| 51 | GO:0055080~cation homeostasis | 8 | 2.225915261 |
| 52 | GO:0001944~vasculature development | 7 | 2.21926412 |
| 53 | GO:0001775~cell activation | 8 | 2.218159459 |
| 54 | GO:0009792~embryonic development ending in birth or egg hatching | 9 | 2.144276154 |
| 55 | GO:0050801~ion homeostasis | 11 | 2.140198475 |
| 56 | GO:0048878~chemical homeostasis | 13 | 2.020496324 |
| 57 | GO:0006812~cation transport | 14 | 2.014594192 |
| 58 | GO:0006952~defense response | 15 | 1.940889527 |
| 59 | GO:0007610~behavior | 11 | 1.866399097 |
| 60 | GO:0042127~regulation of cell proliferation | 16 | 1.61781897 |
| 61 | GO:0007155~cell adhesion | 14 | 1.591529412 |
| 62 | GO:0007186~G-protein coupled receptor protein signaling pathway | 21 | 1.488072914 |
